# LGR4 deficiency results in delayed puberty through impaired Wnt/**β**-catenin signaling

**DOI:** 10.1172/jci.insight.133434

**Published:** 2020-06-04

**Authors:** Alessandra Mancini, Sasha R. Howard, Federica Marelli, Claudia P. Cabrera, Michael R. Barnes, Michael J.E. Sternberg, Morgane Leprovots, Irene Hadjidemetriou, Elena Monti, Alessia David, Karoliina Wehkalampi, Roberto Oleari, Antonella Lettieri, Valeria Vezzoli, Gilbert Vassart, Anna Cariboni, Marco Bonomi, Marie Isabelle Garcia, Leonardo Guasti, Leo Dunkel

**Affiliations:** 1 Centre for Endocrinology, William Harvey Research Institute, Barts and the London School of Medicine and Dentistry, Queen Mary University of London, London, United Kingdom.; 2 Department of Clinical Sciences and Community Health, University of Milan, Milan, Italy.; 3 IRCCS Istituto Auxologico Italiano, Department of Endocrine and Metabolic Diseases and Lab of Endocrine and Metabolic Research, Milan, Italy.; 4 Centre for Translational Bioinformatics, William Harvey Research Institute, and; 5 NIHR Barts Cardiovascular Biomedical Research Centre, Barts and The London School of Medicine and Dentistry, Queen Mary University of London, London, United Kingdom.; 6 Centre for Integrative Systems Biology and Bioinformatics, Department of Life Sciences, Imperial College London, London, United Kingdom.; 7 Université Libre de Bruxelles, Bruxelles, Belgium.; 8 St George’s NHS Foundation Trust, London, United Kingdom.; 9 Children’s Hospital, Helsinki University Hospital and University of Helsinki, Helsinki, Finland.; 10 Department of Pharmacological and Biomolecular Sciences, University of Milan, Milan, Italy.

**Keywords:** Endocrinology, Reproductive Biology, G-protein coupled receptors, Molecular genetics, Neuroendocrine regulation

## Abstract

The initiation of puberty is driven by an upsurge in hypothalamic gonadotropin-releasing hormone (GnRH) secretion. In turn, GnRH secretion upsurge depends on the development of a complex GnRH neuroendocrine network during embryonic life. Although delayed puberty (DP) affects up to 2% of the population, is highly heritable, and is associated with adverse health outcomes, the genes underlying DP remain largely unknown. We aimed to discover regulators by whole-exome sequencing of 160 individuals of 67 multigenerational families in our large, accurately phenotyped DP cohort. *LGR4* was the only gene remaining after analysis that was significantly enriched for potentially pathogenic, rare variants in 6 probands. Expression analysis identified specific *Lgr4* expression at the site of GnRH neuron development. LGR4 mutant proteins showed impaired Wnt/β-catenin signaling, owing to defective protein expression, trafficking, and degradation. Mice deficient in *Lgr4* had significantly delayed onset of puberty and fewer GnRH neurons compared with WT, whereas *lgr4* knockdown in zebrafish embryos prevented formation and migration of GnRH neurons. Further, genetic lineage tracing showed strong Lgr4-mediated Wnt/β-catenin signaling pathway activation during GnRH neuron development. In conclusion, our results show that *LGR4* deficiency impairs Wnt/β-catenin signaling with observed defects in GnRH neuron development, resulting in a DP phenotype.

## Abstract 2

Defects of LGR4/Wnt-β-catenin activity compromise the development of the GnRH neuroendocrine network, resulting in delayed onset of puberty in humans and mice.

## Graphical abstract

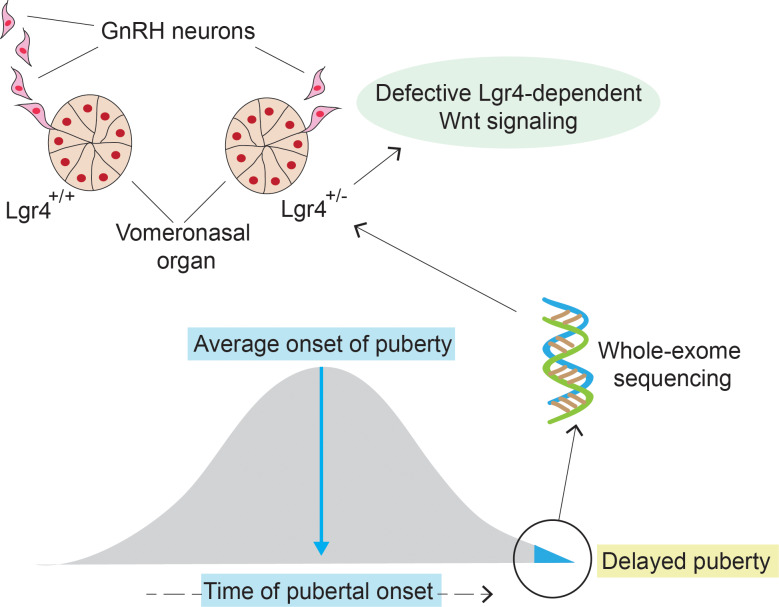

## Introduction

Puberty and the timing of puberty onset are dependent on an intact network of gonadotropin-releasing hormone (GnRH) neurons working together with their afferent and efferent neural and glial connections. Development of this GnRH neuroendocrine network requires a coordinated and timely migration of neurons from the vomeronasal organ (VNO) in the nose to the hypothalamus during embryonic life. We have previously demonstrated that dysregulation in the migratory process leads not only to GnRH deficiency but also to self-limited delayed puberty (DP) (1, 2).

A multitude of factors, important for GnRH neuronal migration and differentiation and hypothalamic and pituitary development, are required for the correct organization of this system. As part of the search for understanding these key influences, large GWASs of age at menarche have identified signals in or near several candidate genes with relevance to forebrain development and function, including *POU1F1*, *TENM2*, and *FRS3* and signals representing *cis-*expression quantitative trait loci for leucine-rich repeat–containing G protein–coupled receptor 4 (*LGR4*) (3). *LGR4* was also identified as a candidate gene for the genetic regulation of pubertal timing in an additional GWAS, which found 1 rare nonsense variant to be associated with the late onset of menarche, low levels of testosterone, and low bone mineral density (4). *LGR4* encodes a receptor for R-spondins, the activation of which potentiates the canonical Wnt signaling pathway. Additionally, it is involved in the development of various organs, including the eyes, liver, reproductive tract, and bone (5). Notably, *LGR4* mutations have not been shown previously to be causal in human disease.

DP affects up to 2% of the population and is associated with adverse health outcomes (6, 7). Self-limited DP (also known as constitutional delay of puberty) is defined as the absence of testicular enlargement in boys or breast development in girls at an age that is 2–2.5 SD later than the population mean (8). Self-limited DP is often familial and is highly heritable, most commonly seen with an autosomal dominant inheritance pattern, indicating the importance of genetic regulation in this phenotype (9). However, for most patients with DP the pathogenic mechanism and genetic basis of their condition remains unknown. We aimed to investigate if defects in further pathways regulating GnRH neuronal migration and development could lead to DP onset in our large, accurately phenotyped cohort of patients with DP. Using a combination of genetic, in silico, in vitro, and in vivo approaches, we have identified that defects in Lgr4 disrupt Wnt/β-catenin signaling appear to affect the development of the GnRH neuronal network, and lead to a phenotype of disrupted pubertal onset in mice and humans.

## Results

### Exome sequencing of families with self-limited DP identifies potentially pathogenic variants in LGR4.

**Figure 1:**
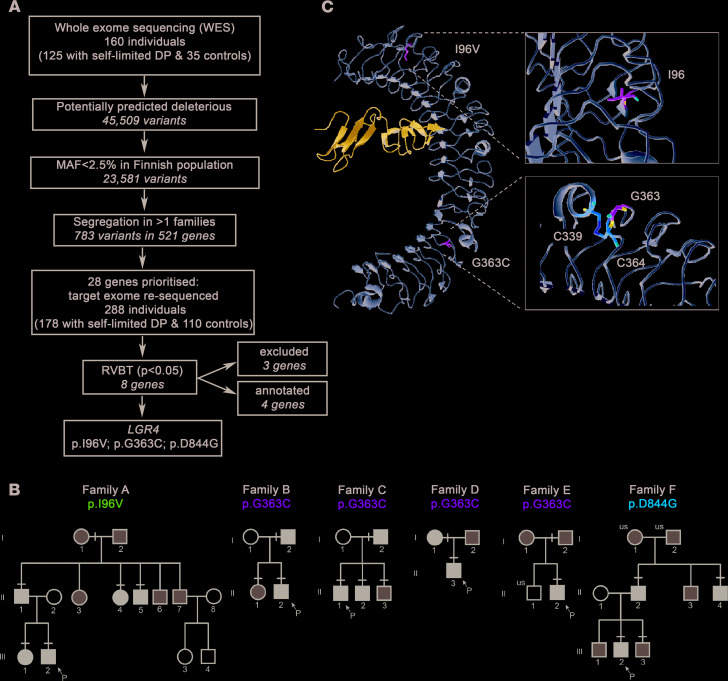
Identification of *LGR4* as a candidate gene for self-limited DP with rare pathogenic variants in patients. (**A**) Whole-exome sequencing (WES) was performed on 160 individuals from our cohort (125 with self-limited DP and 35 controls). Variants were filtered using filters for quality control, predicted functional annotation, minor allele frequency (MAF), and for genes with variants in multiple families. A total of 28 genes were prioritized and were targeted exome sequenced in additional 288 individuals. Further analysis identified genes significantly enriched for pathogenic variants via whole gene burden testing, and genes involved in GnRH neuronal development and puberty timing (1, 2, 10, 11). Excluded, owing to the presence of variants in multiple controls. (**B**) Squares and circles indicate male and female family members, respectively. Black symbols represent affected individuals, gray symbols represent unknown phenotype, and clear symbols represent unaffected individuals. “P” indicates the proband in each family, and “us” indicates unsequenced owing to lack of DNA. A black line above an individual’s symbol indicates heterozygosity for that mutation as confirmed by either WES or Fluidigm array, and verified by Sanger sequencing. (**C**) LGR4 extracellular domain (gold) with variants bound to R-spondin1 (blue). Variants p.I96V and p.G363C are presented (green). p.I96V and p.G363C are in the variable region of LRR2 and LRR12, respectively. p.G363C occurs in close proximity to a cysteine bond (C339-C364; orange), and this substitution introduces a steric clash. p.D844G is within the cytoplasmic domain, and no experimental structure for the LGR4 cytoplasmic domain was available. DP, delayed puberty.

Whole and targeted exome sequencing of 67 informative families from our large cohort with self-limited DP identified 8 genes significantly enriched with rare, potentially pathogenic variants by whole gene burden testing of rare variants. These candidates included 4 genes demonstrated previously to be relevant to the pathogenesis of DP (*IGSF10*, *HS6ST1*, *EAP1*, and *FTO*; refs. 1, 2, 10, and 11), 3 genes that were excluded after Sanger sequencing in patients and controls (*LRRIQ3*, *SEC24A*, and *ZNF560*), and the candidate gene *LGR4* ( ENSG00000205213, gene identification number 107515) (Figure 1A).

### Pedigrees with a potentially pathogenic LGR4 variant display an autosomal dominant inheritance pattern and classical self-limited DP.

We identified 3 rare missense variants in *LGR4* (NM_018490.3: c.286A>G (rs757351670) p.Ile96Val; NM_018490.3: c.1087G>T (rs117543292) p.Gly363Cys; and NM_018490.3: c.2531A>G (rs34804482) p.Asp844Gly) in 6 unrelated families (17 affected individuals) from our familial DP cohort. All segregated with the DP trait with the expected autosomal dominant pattern of inheritance (Figure 1B). All 6 probands were male; however, 2 families contained affected females with maternal inheritance demonstrated in both families. All probands had delayed onset of Tanner stage G2 with low serum gonadotropins and serum testosterone (Table 1). Four of the six males had a height standard deviation score of >–2.0, with a markedly delayed bone age at presentation, and a concurrent delay in age of peak height velocity was recorded for 4 of the 6 probands. All of the males had spontaneously attained Tanner stage G4 or more by 18 years of age, excluding hypogonadotropic hypogonadism. None of the affected individuals had other syndromic features, developmental eye conditions, or other known neurological features. However, MRI brain imaging was not undertaken in any of these individuals.

### In silico analysis of LGR4 variants highlights their likely pathogenicity.

LGR4 is a large protein consisting of 17 extracellular leucine-rich repeats (LRRs) together with a 7-transmembrane region (4). Two variants identified are located in the extracellular (p.I96V and p.G363C) domain, and one variant is located in the intracellular (p.D844G) domain (Supplemental Figure 1A; supplemental material available online with this article; https://doi.org/10.1172/jci.insight.133434DS1). All 3 variants affect amino acids that are highly conserved, as revealed by genomic evolutionary rate profiling score and multiple sequence alignment (Supplemental Figure 1B). Two variants (p.Gly363Cys and p.Asp844Gly) are predicted to be deleterious by ≥three-fifths of the main prediction software tools (Supplemental Figure 1C).

In silico analysis of the glycine-to-cysteine change at position 363 (Protein Data Bank: 4KT1, 2.5 Å) revealed that it occurs in the variable region of LRR 12 of the extracellular domain, and that this substitution introduces a steric clash. Moreover, glycine 363 is juxtaposed with cysteine 364, which forms a cysteine bond with cysteine C339. Overall, this amino acid substitution may alter the structural stability of the LGR4 extracellular domain, thus compromising its protein-binding ability.

The aspartic acid-to-glycine substitution at position 844 lies within the cytoplasmic domain and introduces a small neutral residue in place of the large, negatively charged aspartic acid. Overall, this substitution is predicted to have a damaging effect on LGR4 structure and/or function.

The third variant (p.Ile96Val) lies in the variable region of LRR 2 in the extracellular domain. This variant was retained for functional annotation, despite not being predicted by in silico analysis to cause a major structural change to the LGR4 protein, in view of its rarity and perfect segregation in a large pedigree (Figure 1C).

**Figure 2:**
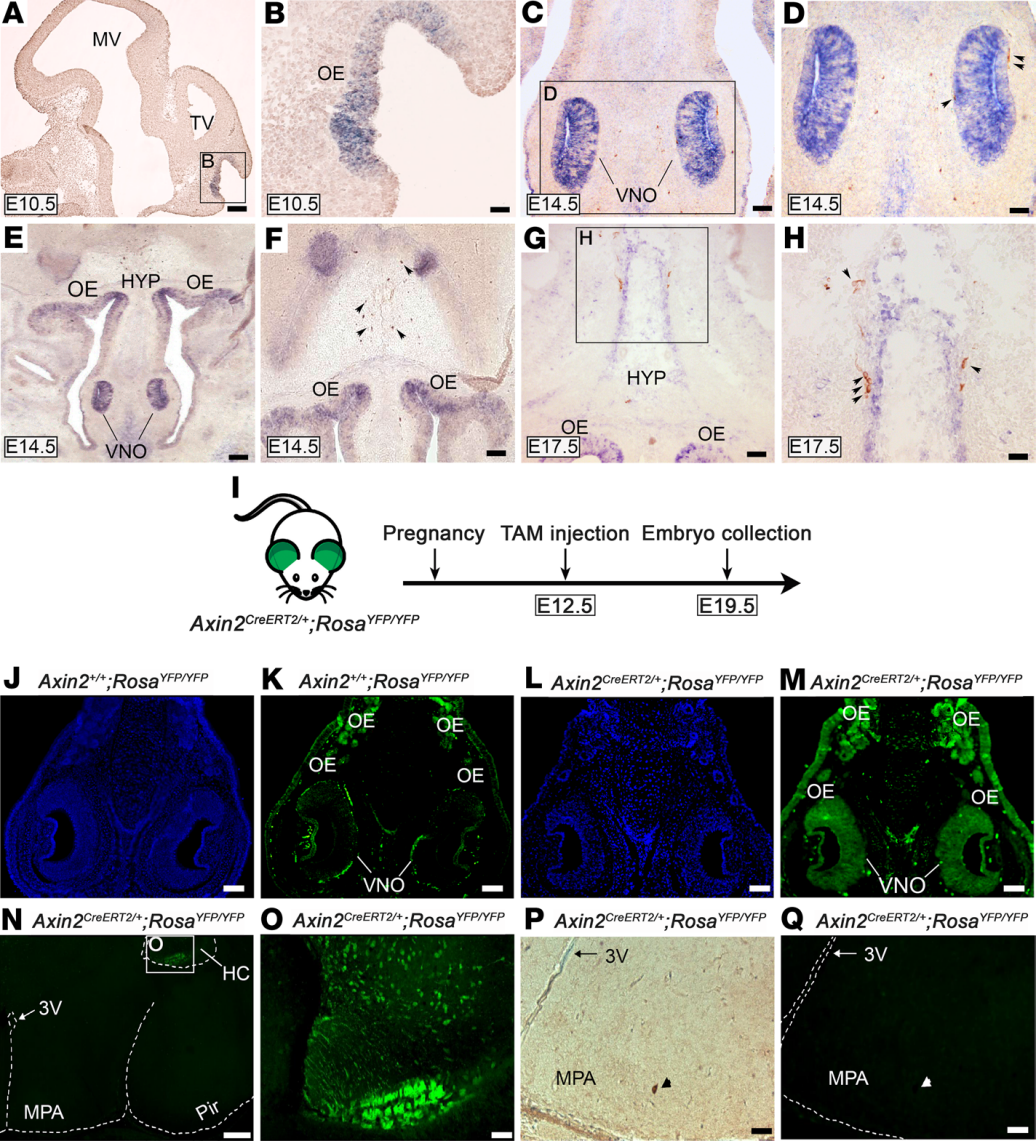
Lgr4*/*Wnt/β-catenin complex is expressed in key regions for GnRH neuronal development and migration. (**A** and **B**) Low- and high-magnification images of in situ hybridization analysis revealing *Lgr4* mRNA localization in the OE at E10.5. (**C** and **D**) At E14.5, the signal is strongly visible in the VNO and immunohistochemistry reveals GnRH neurons exiting the VNO. (**E** and **F**) Low- and high-magnification images showing *Lgr4* mRNA preferentially localized in the VNO, OE, and nuclei of the forebrain at E14.5. (**G** and **H**) Low- and high- magnification images showing *Lgr4* expression at E17.5 in the OE and in an area of the HYP where GnRH neurons (brown) are migrating alongside. (**I**) Schematic showing the mouse model employed for lineage tracing. (**J** and **K**) *Axin2^+/+^ Rosa^YFP/YFP^* control mice reveal a faint and not specific staining for Axin2-GFP. (**L** and **M**) In *Axin2^CreERT2/+^ Rosa^YFP/YFP^* embryos, Axin2-GFP-positive cells are expressed in the VNO and OE, indicating the presence of Wnt/β-catenin responsive cells. (**N** and **O**) Low- and high-magnification images of same specimen showing GFP-positive cells located exclusively in the HC, whereas the MPA is negative. (**P**) Immunohistochemical detection of GnRH neurons followed by (**Q**) immunofluorescence for GFP in *Axin2^CreERT2/+^ Rosa^YFP/YFP^* embryos. A representative GnRH neuron is GFP negative. Scale bars: 25 μm (**A**–**H)**, 100 μm (**J**–**M)**, 250 μm (**N)**, 50 μm (**O**–**Q)**. Representative images of experiments were performed at least 3 independent times. Arrowheads point to GnRH neurons. 3V, third ventricle; E, embryonic day; HC, hippocampus; HYP, hypothalamus; MPA, medial preoptic area; MV, mesencephalic vesicle; OE, olfactory epithelium; Pir, piriform cortex; TV, telencephalic vesicle; VNO, vomeronasal organ. (**A** and **B**) Sagittal sections; (**C**–**Q**) coronal sections.

*Lgr4 is expressed in key areas responsible for GnRH neuronal development and acts through Wnt/β-catenin signaling*. *Lgr4* mRNA is strongly expressed in the adult mouse in cartilage and bone, kidney, adrenal gland, and testis, and at a lower intensity in many other organs. In the mouse embryo, *Lgr4* expression is seen in the VNO and olfactory epithelium (OE) as well as in the eyes, ribs, and esophagus (12). Using in situ hybridization we detected marked expression of *Lgr4* mRNA in the VNO, OE, and at the level of the ventral hypothalamus of the developing mouse embryo (Figure 2, A–H, and ref. 12), suggesting a potential role in the development or migration of GnRH neurons. *Lgr4* mRNA was detected early in development, at E10.5 in the anlage of the OE (Figure 2, A and B). GnRH neurons were seen adjacent to the VNO at E14.5 (Figure 2, C and D) in a strongly *Lgr4*-positive milieu, and also migrating alongside *Lgr4*-positive ventral hypothalamic cells (Figure 2, G and H).

At E12.5, GnRH neurons that had exited the VNO into the nasal mesenchyme were found not to express *Lgr4*, using combined immunofluorescence and in situ hybridization, although some potential co-expression within the VNO in GnRH progenitor cells or early neurons cannot be excluded (Supplemental Figure 2, A–I).

Lgr4 is known to act via Wnt/β-catenin signaling (5, 13); therefore, to examine the activity of this pathway during GnRH neuronal development and migration we used an *Axin2^creERT2/+^ Rosa^YFP/YFP^* mouse model, which is a reporter line known to reliably act as a readout for Wnt-responsive cells (14). We administered tamoxifen to pregnant females at E12.5 and examined embryos at E19.5, when GnRH neurons have reached the hypothalamus (Figure 2I). We found a strong signal in the VNO and OE, exactly matching the expression pattern of *Lgr4* (negative control: Figure 2, J–M). Interestingly, all GnRH neurons migrating to, or within, the hypothalamus were found to be GFP negative (Figure 2, N–Q), whereas the hippocampus (HC, Figure 2, N and O) was strongly GFP positive, as previously reported (14). These data demonstrate that *Lgr4* is strongly expressed in the VNO, a key region responsible for GnRH neuronal differentiation and a hub for Wnt/β-catenin activity, and that nascent GnRH neurons located in the VNO might be affected by responsive Wnt/β-catenin signaling. Taken together, this points to a potential role of Lgr4 in GnRH neuronal development, or in their exit from the VNO to begin migration.

**Figure 3:**
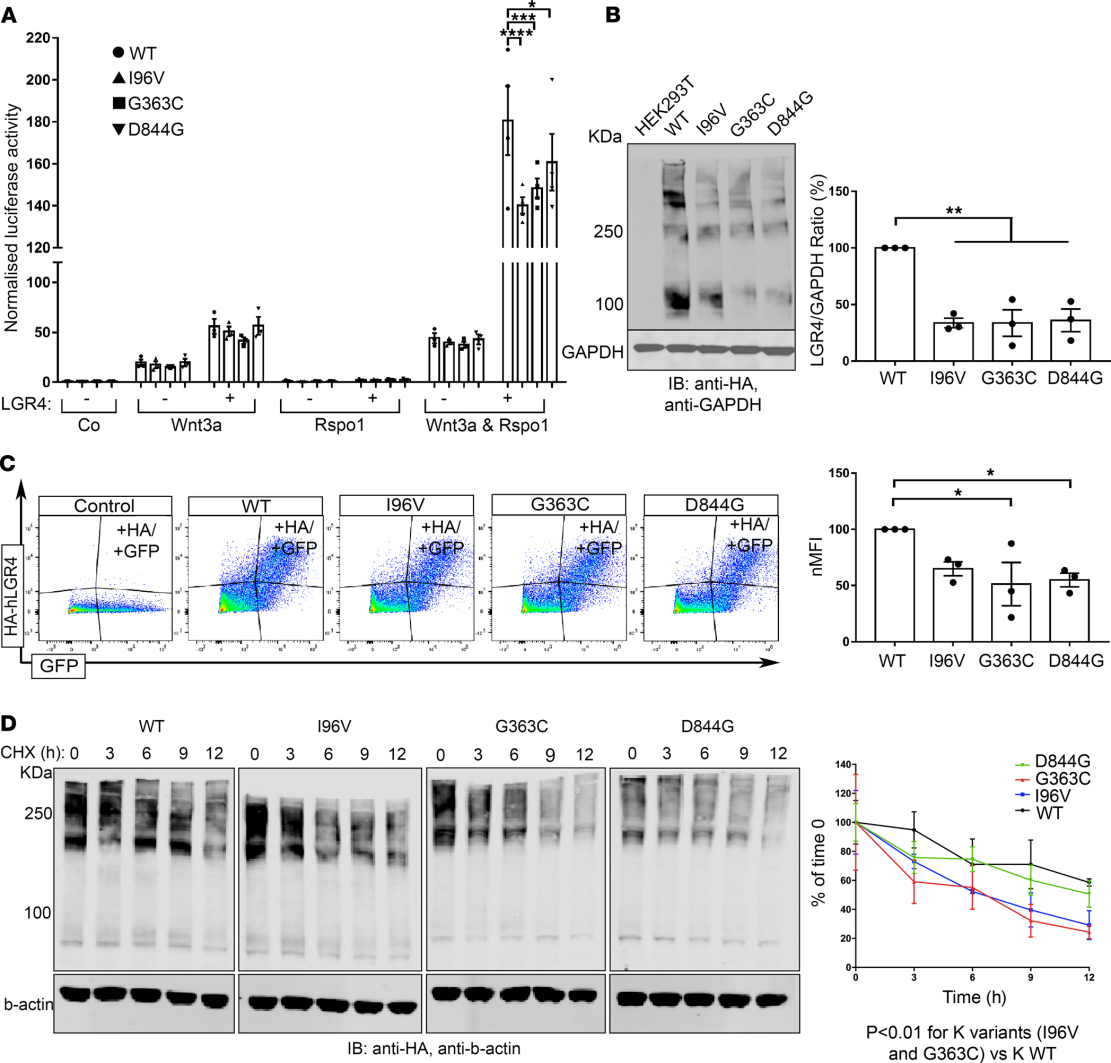
LGR4 mutant receptors affect Wnt/β-catenin signaling owing to defects in protein production, trafficking, and protein turnover. (**A**) HEK293T were nontransfected (–) or transfected (+) with HA-hLGR4 plasmids (WT or mutants; 200 ng/well) and reporter vectors (TOP-Flash and Renilla [150 ng/well]). Signaling was activated with conditioned media treatment, and each transfection normalized by cotransfection with Renilla. HA-hLGR4 mutant receptors resulted in significant reduced luciferase activity. ****P < 0.0001, ***P = 0.004, *P = 0.0437; *n* = 4. (**B**) Western blot and densitometry analysis revealed reduced levels of LGR4 mutants. GAPDH was used as loading control. Molecular weight (KDa) of a protein standard is reported (left panel: WT vs. I96V **P = 0.0054; WT vs. G363C **P = 0.0039; WT vs. D844G **P = 0.0049); *n* = 3. (**C**) Representative plots and quantification of flow cytometry analysis of cell surface expression of WT and mutant LGR4 proteins expressed in HEK293T. Normalized median fluorescence intensity (nMFI) reveals reduced levels of mutant receptors at the plasma membrane compared with HA-hLGR4 WT (WT vs. p.G363C *P = 0.0228; WT vs. p.D844G *P = 0.0498). Control: HEK293T transfected with pcDNA3.1EGFP vector only. *n* = 3. (**D**) LGR4 mutants have a shorter half-life: transiently transfected HEK293T with HA-hLGR4 WT or mutant constructs treated with CHX (50 μg/mL) for different time periods (0, 3, 6, 9, and 12). Levels of LGR4 WT and mutant proteins were expressed relative to untreated LGR4 WT or mutant proteins (0 h); *n* = 4. Statistical analysis by 1-way ANOVA. Half-life was analyzed via a 1-phase decay equation, and degradation speed (K) compared between each mutant and WT protein using the extra sum-of-squares F test. Co, control medium; Rspo1, Rspondin-1.

*LGR4 mutant receptors impair Wnt/β-catenin signaling*. To measure the function of LGR4 variants through Wnt/β-catenin signaling, we used a TOP-Flash luciferase reporter assay. HEK293T cells were transiently transfected with reporter and WT or mutated LGR4 constructs (p.Ile96Val = I96V; p.Gly363Cys = G363C; p.Asp844Gly = D844G). Upon activation of the pathway with conditioned media (Wnt3a and Rspol), all 3 LGR4 mutants had a significantly reduced ability to activate canonical Wnt/β-catenin signaling as compared with LGR4 WT normalized luciferase activity (180.6 ± 16.4) versus I96V (140.2 ± 3.9, *P* < 0.0001), versus G363C (148.3 ± 4.7, *P* = 0.0004), versus D844G (160.8 ± 13.5, *P* = 0.043) (Figure 3A).

With the aim of clarifying the molecular mechanisms underlying this impaired signaling activity, we first analyzed the expression levels of LGR4 WT and mutated proteins. Western blot analysis of HA-tagged human LGR4 (HA-hLGR4) proteins revealed multiple specific bands at different molecular weights: 1 band at approximately 100 KDa, matching the expected molecular weight of LGR4 protein (104 KDa), and multiple additional bands at higher molecular weights, as previously reported (15). HA-hLGR4 mutated protein normalized levels were significantly reduced (Figure 3B, densitometric analysis), suggesting defective protein production caused by all 3 LGR4 mutations. In comparison to WT (100%), mutant LGR4 protein levels were reduced to 33.7% ± 4.2% (*P* = 0.0011), 33.7% ± 11.7% (*P* = 0.0011), and 36.0% ± 10.0% (*P* = 0.0014) for the I96V, G363G, and D844G variants, respectively. These data were corroborated by flow cytometry analysis of plasma membrane expression of LGR4 proteins (Figure 3C). In comparison to WT (100%), the normalized median fluorescent intensity (nMFI) of mutated proteins was reduced to 64.9% ± 6.2% (*P* = 0.0049), 51.3% ± 19.2% (*P* = 0.0647), and 59.0% ± 9.3% (*P* = 0.0117) for the I96V, G363C, and D844G variants, respectively.

We also compared the half-life of WT and mutant LGR4 proteins in HEK293T cells by treating transfected cells with cycloheximide (CHX) at different time points (0, 3, 6, 9, and 12 hours). Although the intracellular variant (D844G) had a half-life (12.89 hours) similar to the WT (15.3 hours), the 2 extracellular variants (I96V and G363C) had a significantly shortened half-life (6.7 and 5.9 hours, respectively) compared with the WT protein (Figure 3D), as also confirmed by the difference in degradation speed (K) of the WT protein and that of the 2 mutant proteins, I96V (*P* = 0.0008) and G363C (*P* = 0.0042). Together, these data demonstrate that these LGR4 mutations result in lower protein expression levels as a whole and in the plasma membrane, and that 2 mutants also had a faster protein turnover. These factors are likely to both contribute to the lower activation of Wnt/β-catenin observed.

### Lgr4^+/–^ mice have a significantly delayed onset of puberty and reduced number of GnRH neurons.

**Figure 4:**
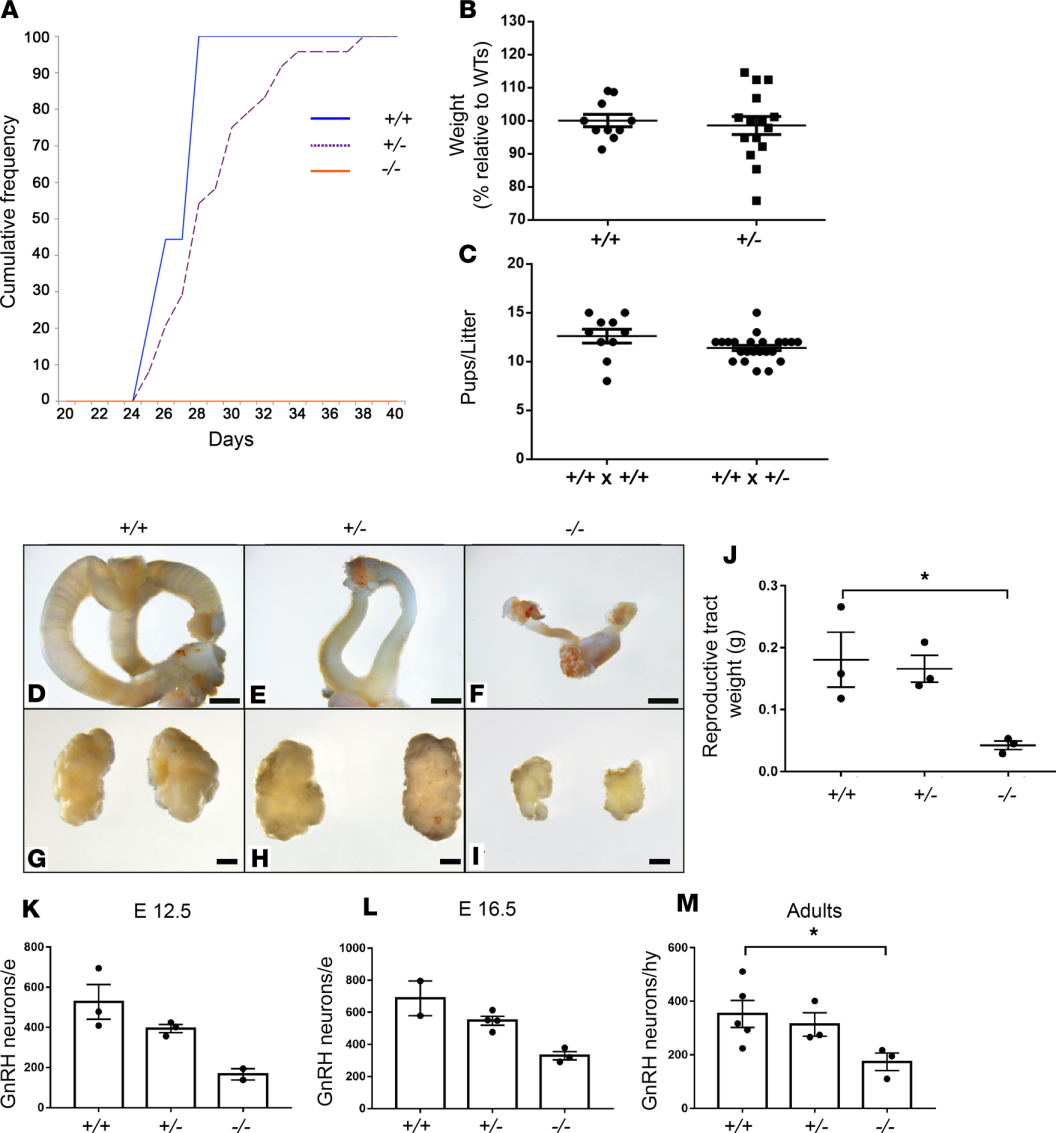
*Lgr4^–/–^* female mice fail to enter puberty and show reduced number of GnRH neurons during fetal and adult life. (**A**) Vaginal opening for pubertal onset shows that *Lgr4^+/–^* mice have a significant (*P = 0.01) delayed onset of puberty compared with *Lgr4^+/+^*. *Lgr4^–/–^* female mice fail to enter puberty completely. *Lgr4^+/+^ n* = 10, *Lgr4^+/–^* *n* = 23, *Lgr4^–/–^ n* = 7. (**B**) *Lgr4^+/–^* mice are not smaller than *Lgr4^+/+^*, as shown by the percentage relative to *Lgr4^+/+^*. *Lgr4^+/+^, n* = 10; *Lgr4^+/–^*, *n* = 15. (**C**) Litter size is not affected when pairing *Lgr4^+/+^* with *Lgr4^+/–^*. Crosses between *Lgr4^+/+^* × *Lgr4^+/+^* and *Lgr4^+/+^* × *Lgr4^+/–^* harbor a normal number in litter size, proving that fertility is not affected in *Lgr4^+/–^* males and females. *Lgr4^+/+^, n* = 10; *Lgr4^+/–^*, *n* = 23. (**D**–**F**) Show gross anatomy of *Lgr4^+/+^*, *Lgr4^+/–^*, and *Lgr4^–/–^* reproductive tracts, respectively. (**G**–**I**) Show gross anatomy of *Lgr4^+/+^*, *Lgr4^+/–^*, and *Lgr4^–/–^* ovaries, respectively. (**J**) *Lgr4^–/–^* females show reproductive tracts significantly reduced in weight compared with *Lgr4^+/+^* females (*P = 0.0366); *n* = 3. Scale bars: 2.5 mm (**D**–**F**), 1 mm (**G**–**I**). (**K**–**M**) GnRH neurons number in E12.5, E16.5, and adults, respectively. In **K**, *Lgr4^+/+^* = 3, *Lgr4^+/–^* = 3, *Lgr4^–/–^* = 2; in **L**, *Lgr4^+/+^* = 2, *Lgr4^+/–^* = 4, *Lgr4^–/–^* = 3; in **M**, *Lgr4^+/+^* = 5, *Lgr4^+/–^* = 3, *Lgr4^–/–^* = 3. In all groups, *Lgr4^–/–^* mice show a reduced number of GnRH neurons compared with *Lgr4^+/+^*. (Adults, *P = 0.0265). *Lgr4^+/–^* mice show a similar trend although not statistically significant. Unpaired 2-tailed *t* test was used for statistical analysis for (**A**–**C**) and (**J**). Kruskal-Wallis test was used for **M**.

To test the hypothesis that heterozygous *Lgr4* deficiency is sufficient to cause DP in vivo, we compared the timing of puberty in *Lgr4****^+/–^*** and *Lgr4^+/+^* female mice by identifying the day of vaginal opening (VO), a proxy measurement for pubertal onset in mice (16). We found that VO was delayed on average by 2.06 ± 1.3 days (*P* = 0.0097) in *Lgr4* *^+/–^* compared with *Lgr4^+/+^* females (Figure 4A); at the time of VO, the *Lgr4^+/–^* and *Lgr4^+/+^* female mice had similar body weights (Figure 4B). Despite DP, the fertility of young adult *Lgr4*^+/–^ mice of both sexes appeared normal, because they seared litters without obvious delay and litters were of similar size to WT mice at 4–6 months of age (Figure 4C). In agreement with the ability to mother litters of normal size, gonadal size was similar in young adult *Lgr4*^+/–^ and *Lgr4*^+/+^ female mice (Figure 4, D–J). Taken together, these findings suggest — consistent with the human phenotype — that *Lgr4* haploinsufficiency delays puberty without compromising fertility in the adult. In contrast, *Lgr4*^–/–^ mice failed to enter puberty entirely and demonstrated substantially reduced gonadal size (Figure 4, A and D–J).

In view of the evidence that Lgr4 may affect GnRH development or migration, we next assessed the number of GnRH neurons in the nasal placode region during early embryogenesis, and in the hypothalamus in later embryogenesis and in postnatal mice. The number of GnRH neurons was markedly decreased in the *Lgr4* ^–/–^ mice as compared with WT. At E12.5, E16.5, and adult age, *Lgr4* ^–/–^ mice presented a reduction of GnRH neurons of 68.5% ± 28%, 52.0% ± 25.9%, and 50.7% ± 32.6%, respectively, as compared with *Lgr4*^+/+^ (Figure 4, K–M). An intermediate phenotype in GnRH number was observed in the heterozygous *Lgr4*^+/–^ mice at all 3 developmental stages. GnRH neuron morphology appeared unchanged in the heterozygous and homozygous mice as compared with WT (Supplemental Figure 3, A–I). Weight and gross anatomy of the brain were not significantly different between the 3 groups (Supplemental Figure 4, A–D).

### Knockdown of lgr4 in zebrafish embryos impairs gnrh3 neuronal development.

**Figure 5:**
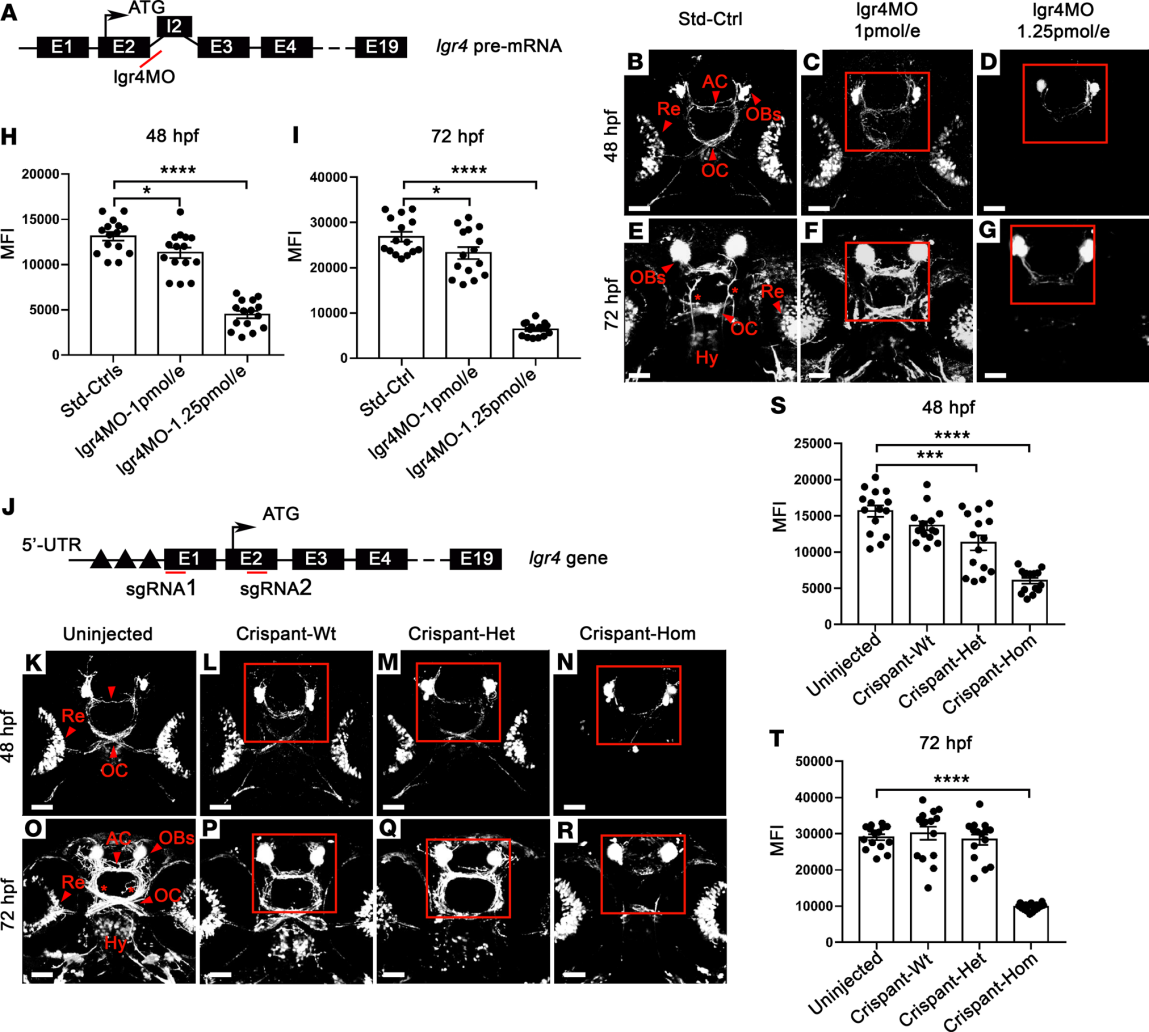
Lgr4 impairs GnRH3-neuron development in morphants and Crispants Zebrafish. (**A**) Morpholino-mediated (MO-mediated) knockdown of the lgr4 gene in zebrafish: representation of lgr4 pre-mRNA. The lgr4MO targeted the exon2-intron2 boundary (red line). (**B**–**G**) Live-imaging acquisition of GnRH3 neurons in lgr4 morphants. (**B** and **E**) normal development of GnRH3 neurons at 48 and 72 hpf. Strong GnRH3 signal is visible at the level of the OBs, AC, along the OC and Re. At 72 hpf, GnRH3 hypothalamic (Hy) projections (asterisks) are also detectable. (**C** and **F**) embryos injected with 1 pmol/embryo. (**D** and **G**) embryos injected with 1.25 pmol/embryo. Images are acquired in ventral view, anterior to the top. (**H** and **I**) Quantification of the mean fluorescence intensity (MFI) using ImageJ Software. A significant reduction in MFI was observed in morphants vs. ctrl: at 48 hpf, lgr4MO 1 pmol/embryo *P = 0.0268; lgr4MO 1.25 pmol/embryo ****P < 0.0001; at 72 hpf, lgr4MO 1.25 pmol/embryo *P = 0.0290; lgr4MO 1.25 pmol/embryo ****P < 0.0001; *n* = 15 (**J**) Crispr/Cas-mediated KO of the lgr4 gene in zebrafish: representation of lgr4 gene, including the ATG start site and regulatory regions located in the 5′ UTR (black triangles). Localization of the 2 sgRNAs used (red lines). (**K**–**R**) Live-imaging acquisition of GnRH3 neurons in lgr4 Crispants. (**K** and **O**) uninjected embryos. (**L** and **P**) Crispant-Wt. (**M** and **Q**) Crispant-Het. (**N** and **R**) Crispant-Hom (all at 48 and 72 hpf). (**S** and **T**) A significant reduction in MFI was observed in Crispants vs. ctrl. At 48 hpf, Crispant-Het ***P = 0.0003; Crispant-Hom ****P < 0.0001; at 72 hpf Crispant-Hom ****P < 0.0001; *n* = 15. Red squares indicate region of interest (ROI) used for GnRH3 fiber quantification. Scale bars: 50 μm (**B**–**G** and **K**–**R**). Statistical analysis by 1-way ANOVA.

To further characterize the role of *LGR4* during GnRH neuron development, we investigated knockdown (KD) of *lgr4* in a well-established transgenic *gnrh3*:gfp zebrafish model (17, 18), first using splice site-blocking morpholino (MO) for transient KD (Figure 5A). MO doses (1 pmol/embryo and 1.25 pmol/embryo) were selected because they reduced expression to 50% and < 10% of WT *lgr4* mRNA, thus producing conditions similar to the heterozygous and homozygous state (Supplemental Figure 5, A–C).

At 48 hpf, embryos injected with 1 pmol/embryo displayed a significant reduction in MFI of GnRH3 fibers at the level of the anterior commissure (AC), optic chiasm (OC), and retina (Re) (Figure 5, C and H; MFI: 11310 ± 590.6) in comparison with controls (Figure 5, B and H; MFI: 13132 ± 482.2; *P* = 0.027). Injection of 1.25 pmol/embryo led to a more severe reduction in the formation of GnRH3 fibers (Figure 5, D and H; MFI: 4475 ± 408.3; *P* = 1 × 10^–5^). Similarly, at 72 hpf, migration of GnRH3 axon fibers to the hypothalamus was affected in embryos injected with 1 pmol/embryo lgr4MO (Figure 5, F and I; MFI: 23,250 ± 1285.8) when compared with controls (Figure 5, E and I; MFI: 26,863 ± 1046.5; *P* = 0.03), and more severely disrupted in 1.25 pmol/embryo injected morphants (Figure 5, G and I; MFI: 6366 ± 389.2; *P* = 5 × 10^–7^).

To confirm the effects on GnRH3 system development of *lgr4* inactivation, we used a CRISPR/Cas9 phenotyping protocol that relies on repeated noninvasive live analysis of GnRH3 neuron development in control and Crispant *tg(gnrh3*:gfp) fish. The single-guide RNA (sgRNA) was designed to cause a deletion of a region of about 500 bp containing the transcription start site of the *lgr4* mRNA (Figure 5J and Supplemental Figure 5, D–F). Crispant-Wt displayed normal development of GnRH3 neurons as compared with uninjected embryos at 48 and 72 hpf (Figure 5, K, L, O, and P), as revealed by MFI at 48 hpf (Figure 5S) and at 72 hpf (Figure 5T). Similar to the MO results, at 48 hpf homozygous Crispants (Crispant-Hom) displayed a disorganization of the olfactory bulbs (OBs) with a significant reduction of MFI of GnRH3 fibers at the level of AC, OC, and Re (Figure 5, N and S; MFI: 6052 ± 399.9), as compared with uninjected embryos (Figure 5, K and S; MFI: 15,669 ± 777.9; *P* = 8 × 10^–6^). At 48 hpf, heterozygous Crispants (Crispant-Het) showed a milder, but significant, reduction in GnRH3 neuronal development compared with uninjected embryos (Figure 5, M and S; MFI: 11,297 ± 1035.1; *P* = 0.0003), whereas at 72 hpf no significant changes were observed (Figure 5, Q and T). At 72 hpf, the GnRH3 signal in the AC as well as in the hypothalamic innervations was strongly reduced in Crispant-Hom as compared with uninjected embryos (Figure 5, R and T; uninjected MFI: 29,059 ± 828.7 vs. Crispant-Hom MFI: 9766 ± 261.6; *P* = 2 × 10^–7^). Taken together with the previous results from our study, these data demonstrate that lgr4 is required for regulation of the development of the GnRH neuronal system in mouse, zebrafish, and humans.

## Discussion

DP can be a clinical presentation of many different pathological mechanisms. Recent evidence has demonstrated that there may be a fetal origin of disorders of pubertal timing, with deficiency in key genes that govern the development of the gonadotropin releasing hormone system resulting in a spectrum of conditions ranging from isolated DP to absent puberty with anosmia (19–21). However, the genetic basis of DP remains largely unclear. We aimed to further explore the hypothesis that defects of GnRH neuronal development could present with a DP phenotype in adolescence, with subsequent normal reproductive capacity.

In this study, we have identified 3 deleterious mutations in *LGR4* in 6 unrelated pedigrees with self-limited DP, and describe the mechanistic basis for their pathogenicity and influence on the timing of pubertal onset. Lgr4 is known to modulate the Wnt/β-catenin signaling cascade and to be important for development and stem cell survival (22). It is thought to act, once coupled to its ligand R-spondin, to prevent Wnt receptor degradation via inhibition of ubiquination by Rnf43 and Znrf3 (23). *LGR4* had already been implicated as a regulator of pubertal timing through GWAS of age at menarche in women and age of voice break in men (4, 7); however, mutations in *LGR4* had not previously been demonstrated to be causal in human disease.

Rare pathogenic variants in *LGR4* were enriched in our self-limited DP cohort as compared with control populations, and these mutations were inherited in the recognized autosomal dominant pattern seen in this condition (9, 24). The affected members of these families displayed typical self-limited DP, with puberty onset having commenced before 18 years of age. None of the affected individuals had neurological or other associated phenotypic abnormalities (25). The variants identified all led to functional impairment of LGR4 protein ability to activate Wnt signaling, as demonstrated by luciferase reporter assay. All 3 resulted in decreased protein expression, owing to reduced cell surface expression, faster degradation, or both, pointing to a reduced protein bioavailability accounting for the DP phenotype (26). Interestingly, the 3 mutations are found in different domains in the LGR4 protein, 2 within LRR regions of the extracellular domain, and 1 within the cytoplasmic domain. Complementing previous studies, we have shown that *LGR4* is highly expressed in the VO, OE, and hypothalamus of the developing mouse (12).

To investigate the role *LGR4* plays in pubertal timing in vivo, we used 2 different murine models. First, lineage-tracing analysis identified activation of the Wnt/β-catenin pathway in embryonic regions with strong *Lgr4* expression, including the VNO and OE. The observation that hypothalamic GnRH neurons were descendant of cells not endowed with endogenous Wnt/β-catenin activity suggests the intriguing possibility that nascent and/or migrating GnRH neurons might be affected by Lgr4 signaling by adjacent cells in a paracrine fashion. Second, using a *Lgr4* KO mouse model we were able to determine that *Lgr4* deficiency results in reduced numbers of GnRH neurons at both early migratory stages and within the hypothalamus. Corroborating data from confocal evaluation of transgenic gnrh3:gfp zebrafish also point to the relevance of *lgr4* during the early development of GnRH3 neurons.

Previous data show that a functional redundancy exists within the GnRH neuronal population (27), thus *LGR4* deficiency, via defective Wnt/β-catenin signaling, may not only reduce GnRH neuronal number but also affect the function of these neurons within the hypothalamic neurosecretory network. This hypothesis requires future investigation beyond the scope of this manuscript.

The DP phenotype seen in our patients with heterozygous *LGR4* mutations was recapitulated in the *Lgr4*^+/–^ mice, which demonstrated late VO, a pragmatic proxy measure of puberty onset. Like our patients, these mice had no reduction in reproductive capacity postpubertally, as evidenced by comparable gonadal size, morphology, and fertility to WT animals, and were otherwise healthy and of normal body weight.

In contrast, *Lgr4*^–/–^ mice had complete failure to enter puberty with a marked reduction in GnRH neuronal number at all stages; in keeping with this, *Lgr4*^–/–^ mice had markedly underdeveloped gonadal structures, similar to other rodent models of GnRH deficiency (28, 29), which in part may be attributed to a direct role of LGR4 in gonadal development (30, 31). KD of *lgr4* in developing zebrafish also led to significant defects with GnRH neuronal development and morphology. Mutations in *LGR4* have not been identified in patients with conditions of GnRH deficiency such as hypogonadotropic hypogonadism. Notably, there are no individuals homozygous for complete loss-of-function *LGR4* mutations found in the gnomAD database. This, taken with the early mortality and organ dysfunction seen in the KO mouse, suggests that loss-of-function of *LGR4* leading to complete GnRH deficiency would result in a very severe phenotype, possibly incompatible with life.

Significantly delayed onset of puberty is frequently seen in the pediatric clinic because it affects up to 2% of the population. Accurate diagnosis has potential health and economic impacts, as late puberty is associated with adverse outcomes, including decreased bone mineral density, osteoporosis (32, 33), psychological distress (34, 35), and poor overall health (6, 36). A clear understanding of the genetic control of pubertal timing will aid diagnosis, which can be difficult and require prolonged, expensive investigation in these adolescent patients, and optimize management in this patient group.

In summary, here we have used a combination of next-generation sequencing methods with RNA expression analysis, cell culture, and animal models to identify that defects of *LGR4*-Wnt/β-catenin activity are associated with compromised development of the hypothalamic GnRH neuroendocrine network and result in delayed onset of puberty in humans and mice.

## Methods

### Patients

The large cohort of individuals (*n* = 910) with self-limited DP studied here has been described in previous reports from our group (1, 2, 9). In brief, this cohort includes patients with self-limited DP (*n* = 492), defined as the onset of Tanner genital stage II (testicular volume > 3 mL) >13.5 years in boys or Tanner breast stage II > 13.0 years in girls (i.e., 2 SD later than average pubertal development) (8) and their unaffected relatives. Probands had been managed with specialist pediatric units in Finland between 1982 and 2004. All affected individuals met the diagnostic criteria for self-limited DP, with chronic illness as a cause for functional hypogonadotropic hypogonadism excluded by medical history, clinical examination, and biochemical investigations. Congenital or acquired hypogonadotropic hypogonadism, if suspected, was excluded by spontaneous pubertal development by 18 years of age at follow-up.

### Genetic analysis

A total of 67 probands with self-limited DP, selected from those families in the cohort with the greatest number of affected individuals (male, *n* = 57; female, *n* = 10), 58 affected family members (male, *n* = 36; female, *n* = 22), and 35 of their unaffected family members (male, *n* = 13; female, *n* = 22), underwent initial genetic analysis. This involved whole-exome sequencing of DNA extracted from peripheral blood leukocytes of these 160 individuals using a Nimblegen V2 or Agilent V5 platform and Illumina HiSeq 2000 sequencing. The exome sequences were aligned to the UCSC hg19 reference genome using the Burrows-Wheeler Aligner software (BWA-MEM [bwa-0.7.12]). Picard tools [picard-tools-1.119] was used to sort alignments and to mark PCR duplicates. We used the Genome Analysis Toolkit (version 3.4-46) to realign around indels and recalibrate quality scores using dbSNP, Mills, and 1000 genomes as reference resources. Variant calling and joint genotyping using pedigree information was performed using HaplotypeCaller in GVCF mode from the Genome Analysis Toolkit. The resulting variants were filtered using the variant quality score recalibration (VQSR) function from GATK.

Variants were analyzed for potential causal variants using filters, including for quality control, predicted function, minor allele frequency (MAF) and biological relevance (Figure 1A). A MAF threshold of < 2.5% in the 1000 Genomes database, the NHLBI exome variant server and the ExAC and gnomAD databases was used. A case-control analysis to exclude variants present in more than 1 unaffected control was applied. A multiple family filter to retain only genes with variants present in more than 1 proband was also performed. Targeted exome sequencing (Fluidigm) of the remaining candidate genes was then performed in 42 further families from the same cohort (288 individuals, 178 with DP; male = 106, female = 72 and 110 controls; male = 55, female = 55, Figure 1A), with filtering as in (1).

Whole gene rare-variant burden testing was performed after sequencing. Fisher’s exact test was used to compare the prevalence of deleterious variants in our cohort with the Finnish population, using the ExAC Browser (Exome Aggregation Consortium [ExAC]: accessed September 2015).

### In silico analysis

The amino acid sequence of the human LGR4 was obtained from UniProt (UniProt id Q9BXB1) (37). The LGR4 x-ray structure of the extracellular domain was retrieved from the Protein Data Bank (38). Homology modeling was performed on the intracellular domain by using the in house Phyre2 prediction tool (39). FoldX was used to build the 3D structure of LGR4 mutant proteins and to calculate the difference in free energy between the WT and mutant LGR4 (40). The structural analysis was performed manually. The in silico predictions were obtained from SIFT and Polyphen2 prediction tools (41, 42).

### Site-directed mutagenesis

*LGR4* mutations were inserted in pcDNA3.1/-HA-h*LGR4* (provided by Jim Liu and Xing Gong, Institute of Molecular Medicine, Houston, Texas, USA) using QuikChange II Site-Directed Mutagenesis Kit (Agilent Technologies) following the manufacturer’s instructions. The p.Ile96Val missense mutation was inserted using the following primers: forward 5′-CGACCTTTCTTTTGTCCACCCAAAGGCC-3′ and reverse 5′-GGCCTTTGGGTGGACAAAAGAAAGGTCGT-3′. The p.Gly363Cys missense mutation was inserted using the following primers: forward 5′-TTCCAAGTTTTAATTGTTGCCATGCTCTG-3′ and reverse 5′-CAGAGCATGGCAACAATTAAAACTTGGAA-3′. The p.Asp844Gly mutation was inserted using the following primers: forward 5′-GGATTTCTACTACGGCTGTGGCATGTACT-3′ and reverse 5′-AGTACATGCCACAGCGTAGTAGAAATCC-3′. Vectors were fully sequenced after mutagenesis.

### Cell culture

HEK293T cell line (sourced from ATCC) was cultured in DMEM (MilliporeSigma) supplemented with 10% FBS (Invitrogen) and 1% penicillin/streptomycin solution (Pen/Strep; Invitrogen), referred as complete medium. LWnt-3a cell line (gifted by Bethan Thomas and Francesco Dell’Accio, Translational Medicine and Therapeutics, QMUL, London, United Kingdom) was cultured in DMEM supplemented with 10% FBS and 0.4 mg/mL Geneticin (G-418; Thermo Fisher Scientific). Cell growth media were warmed before contact with cells. All cells were incubated in a humidified incubator at 37°C and 5% CO_2_. Cells were assessed for mycoplasma contamination (MycoAlert Detection Kit, Lonza) on a monthly basis.

### Transfection

Cells were plated at the appropriate density (12.5 × 10^4^ cells/well in a standard 24 well plate, 0.3 × 10^6^ cells/well in a 6-well cell culture plate or 2.2 × 10^6^ cells/dish in a 10 cm^2^ dish). After 24 hours, the medium was replaced to a serum-free DMEM for 1 hour. For a 24-well cell culture plate, a total of 0.5 μg/well DNA was diluted serum-free in DMEM/High Glucose (0.5 mL/well) and 1 mg/mL polyethylenimine (MilliporeSigma). A total of 1 μg/well and 5 μg/dish DNA were used for transfection in 6-well cell culture plates and 10 cm^2^ dishes, respectively. The transfection mixture was gently mixed and incubated at room temperature for 10 minutes, before being added dropwise onto the wells. Three hours after transfection, the medium was replaced with DMEM complete medium. Luciferase assays were performed in 24-well cell culture plates, Rspondin-1 conditioned medium preparation was performed in 10 cm^2^ dishes and every other transfection was performed in 6-well cell culture plates.

### Protein extraction and Western blot analysis

Forty-eight hours after transfection, cells were harvested and lysed in RIPA buffer (MilliporeSigma) supplemented with Protease Inhibitor (Roche Diagnostics Ltd.) for 20 minutes on ice, and samples were centrifuged for 20 minutes at 13,000 rpm at 4°C. The concentration of the supernatant was measured by the BCA kit (Thermo Fisher Scientific) according to the manufacturer’s instructions. Equal amount of proteins was separated by SDS-PAGE (pre-cast 4%–12% polyacrylamide NuPage BisTris gels; Invitrogen) and transferred on nitrocellulose (Promega) membrane. After blocking with 5% nonfat milk in PBS containing 0.1% Tween-20 (PBT) for 1 hour at room temperature, membranes were incubated overnight at 4°C with primary antibodies: rabbit anti-HA (Santa Cruz Biotechnology, sc-805, diluted 1:1000) and mouse anti-glyceraldehyde-3-phosphate dehydrogenase (GAPDH, Santa Cruz Biotechnology, sc-365062, diluted 1:5000), or mouse anti-β-actin (Abcam, ab8226, diluted 1:5000) in PBT. After washes in PBT, the membranes were probed for 1 hour with IRDye 800 goat anti-rabbit and IRDye 680 goat anti-mouse secondary antibody (diluted 1:10,000, Licor). After washes in PBT, membranes were scanned and analyzed using the Odyssey Fc Imaging System (Licor).

### CHX chase analysis for protein half-life

Forty-eight hours after transfection, cells were treated with or without 50 μg/mL CHX (ab120093, Abcam) diluted in complete medium. Proteins were extracted after 0 (no treatment), 3, 6, 9, and 12 hours. Proteins were subjected to Western blot with rabbit anti-HA (Santa Cruz Biotechnologies, sc-805, diluted 1:1000) and mouse anti-β-actin (Abcam, ab8226, diluted 1:5000) antibodies. Densitometry analysis was performed, and the normalized protein levels were converted to percentages, with time = 0 being 100%. HA-LGR4 half-life was determined using a 1-phase decay equation. The results were plotted as curves for each WT or mutant proteins on which half-life of each protein was calculated, using the formula: half-life = ln(2)/K. The degradation speed, represented by the K value, was compared between each mutant protein, and the WT protein using the extra sum-of-squares F test, using GraphPad Prism 7.

### Wnt3a and Rspondin-1 conditioned media production

LWnt-3a cells were passaged 1:10 in 10 mL of DMEM with 10% FBS and 1% pen/strep in T75 and grown for 4 days, approximately to confluence. The first batch of medium was removed and filter sterilized. A total of 10 mL of fresh medium were added, and cells were cultured for a further 3 days. The second batch of medium was collected, filter sterilized, and added to the first batch (1:1). The conditioned medium was stored at 4°C or aliquoted and stored at –20°C for long-term storage. Following a transient transfection of HEK293T cell line with the vector encoding human Rspondin-1 fused to alkaline phosphatase (AP-hRspo1 vector, gifted by Andrey Glinka, Division of Molecular Embryology, Heidelberg, Germany) in 10 cm^2^ dishes, conditioned medium, containing secreted AP-hRspo1, was collected at 24 and 48 hours after transfection, centrifuged to remove any cell debris, and filter sterilized using 0.22 μm filters (Sartorious). The expression of the fusion protein was assessed with Western blot (not shown). The conditioned medium was stored at 4°C or at –20°C for long-term storage.

### TOP-Flash Dual Luciferase Reporter assay

HEK293T cell line was transiently cotransfected with the same amount of TOP-Flash (MilliporeSigma) and SV-40 Renilla (Promega) vectors (150 ng each vector/well of a 6-well plate) in conjunction with 200 ng/well HA-hLGR4 WT or mutated vectors. The total amount of DNA transfected was kept constant to 500 ng/well by adding the appropriate amount of pBlueScript vector. After 24 hours, cells were treated with 500 μL of conditioned medium: control, Wnt3a, Rspo1, or Wnt3a+Rspo1. After a 24-hour treatment, cells were harvested and assayed for luciferase using the Dual Luciferase Reporter System (Promega) following the manufacturer’s instructions. Each experiment was performed in triplicate and repeated 4 independent times. Samples were processed using the POLARstar Omega microplate reader, and data were analyzed using MARS Omega software.

### Flow cytometry

HEK293T cells were cotransfected in a 6-well cell culture plate with 800 ng each vector/well of HA-hLGR4 (WT or mutated) or pBlueScript (negative controls) and pCDNA3-EGFP (200 ng/well; 13031, Addgene). Forty-eight hours after transfection, cells were harvested using Trypsin/EDTA (MilliporeSigma). Cells were washed twice with FACS buffer (PBS, 2% FBS, 2 mM EDTA) and incubated for 30 minutes at 4°C with the primary antibody (rabbit anti-HA, Santa-Cruz, diluted 1:500 in FACS buffer), or left in FACS buffer (negative control). Cells were washed twice in FACS buffer and then incubated with the secondary antibody. Negative controls included Alexa Fluor 568 goat anti-rabbit (Thermo Fisher Scientific) diluted 1:1000 in FACS buffer. Flow cytometry measurements were performed using a BD LSRFortessa flow cytometer, and data were analyzed using FlowJo (v7.63, Tree Star, Inc.). Number of events was kept constant to 10,000/tube. For comparison analysis of the double-positive populations (+HA/+GFP), the nMFI was calculated for the signal emitted by the B530-30 (GFP) and the R670-14 (HA-LGR4) channels, using the following formula: nMFI = (MFI sample)/(MFI control).

### Animal procedures

*Lgr4/Gpr48^Gt* mice containing the LacZ knockin allele at the *Lgr4* locus were on a CD1 background (12, 43). Animals had access to both food and water ad libitum and were housed under controlled conditions of light (12 h light, 12 h dark) and temperature (21°C). Female puberty was examined by daily checking for the onset of vaginal opening. Mice were pooled from 7 different litters. Transgenic mice were genotyped using genomic DNA isolated from tail tissue samples using the following primer sets: *Lgr4* UpA: 5′-CCAGTCACCACTCTTACACAATGGCTAAC-3′, *Lgr4* DownB: 5′-ATTCCCGTAGGAGATAGCGTCCTAG-3′, *Lgr4* DownC: 5′-GGTCTTTGAGCACCAGAGGAC-3′. The 3 primers are used to amplify genomic *Lgr4* WT (805 bp) and mutated (650 bp) regions.

### Tissue processing

Embryos (E10.5-E14.5-E16.5) or dissected adult brains and gonads from C57BL/6 and *Lgr4/Gpr48^Gt* mice were isolated and fixed in 4% paraformaldehyde (MilliporeSigma) in PBS (pH 7.4) overnight at +4°C and washed 3 times in PBS (pH 7.4). Specimens were cryoprotected in 30% sucrose in PBS and frozen in OCT compound (VWR); 20-μm-thick serial coronal sections were collected on Superfrost Plus slides (VWR) for GnRH neurons counting. Serial coronal and sagittal sections (12 μm thick) were collected for tissue expression analysis.

### In situ hybridization and immunohistochemistry

*Lgr4* was PCR-amplified from mouse lungs using the following primers: mLgr4 FOR: 5′-TCTTGTTCATCACTGCCTGC-3′, REV: 5′-AGCTGTCCGAGACAAAGGAA-3′. Amplified cDNAs were cloned into the vector pGEM-T easy (Promega) and linearized with the appropriate restriction enzymes. Probe preparation and in situ protocol were performed as previously (44). When colabeling was desired, after in situ, the sections were incubated with primary antibodies (anti-GnRH, 20075, Immunostar) diluted 1:1000 in PBS–Triton 0.1%, overnight at room temperature (RT) (45). After 3 washes with PBS–Triton 0.1%, the slides were incubated for 2 hours at RT with biotin-conjugated goat secondary antibodies (Vector Laboratories), diluted 1:300 in PBS and, after further washes, with the avidin–biotin complex (ABC staining kit, Vector Laboratories). The sections were reacted with 3,3′-diaminobenzidine (DAB, Vector Laboratories) and mounted in an aqueous compound formed by PBS and glycerol (3:1). Images were acquired using a Leica DM5500B microscope (Leica), equipped with a DCF295 camera (Leica) and DCViewer software (Leica), and then processed with Abode Photoshop CS6 and Adobe Illustrator CS6 software.

### Zebrafish experiments

#### Zebrafish lines and maintenance.

The *tg*(*gnrh3*:gfp) (46) zebrafish embryos were collected by natural spawning, and embryos were raised at 28°C under standard conditions (47) and staged according to hours post fertilization (hpf) as previously described(48). Beginning from 24 hpf, embryos were cultured in fish water containing 0.003% 1-phenyl-2-thiourea (MilliporeSigma) to prevent pigmentation and 0.01% methylene blue to prevent fungal growth (47).

#### Lgr4 KD by MO microinjection.

To generate embryos with transient KD of *lgr4*, different doses (0.5, 1, and 1.25 pmol/embryo) of a specific splice site-blocking MO, designed to cause the retention of the intron 2 during the splicing of the pre-mRNA, were microinjected. An antisense MO (lgr4MO) against the *lgr4* pre-mRNA were synthesized by GeneTools (Philomath). The lgr4MO (5′-TACTGTGGTTACTTACAGGTAGTAG-3′) was designed on the exon2-intron2 boundary. As control for unspecific effects, each experiment was performed in parallel using 1.25 pmol/embryo of a standard control MO (Std_Ctrl), which has no specific target in the zebrafish genome. Morphological evaluation of lgr4MO-injected embryos at 24 hpf showed normal growth with the absence of gross developmental abnormalities in most of the injected embryos (Supplemental Figure 5A). The mean mortality rate was 12.8% in *lgr4* morphants, compared with 8.4% of control embryos, suggesting a low toxicity of the MO injection. By both RT-PCR and qPCR, we observed that *lgr4* was reduced in a dose-dependent manner in embryos injected with 0.5, 1, and 1.25 pmol/embryo lgr4MO, compared with the Std_Ctrls (Supplemental Figure 5, B and C).

#### Lgr4 KO using the CRISPR/Cas9 System.

Two different sgRNA targets were chosen in early exons (Exon1-sgRNA CAAACCGCGACGAAACACGACGG and Exon2-sgRNA GGACTGACCAGCGTCCCCACCGG) to potentially introduce a large deletion that include the ATG starting site and some regulatory regions necessary for mRNA transcription. The sgRNA were generated by in vitro transcription from oligonucleotide-based templates using the MEGAscript T7 Transcription kit (Ambion), as previously described (49).

Injection experiments with different sgRNAs showed that the microinjection of 300 pg Cas9 protein together with 200 pg for each sgRNA resulted in a tolerable toxicity with a mean mortality rate of 9.4%, compared with 5.3% of the uninjected embryos used as a control group. Moreover, the great majority of Crispants displayed normal morphological development at 24 hpf (Supplemental Figure 5D).

Both RT-PCR and qPCR experiments performed in single embryos revealed a high efficiency of gene inactivation. A total of 39% and 43% of Crispants showed monoallelic and biallelic inactivation of the *lgr4* gene, respectively. Lgr4 expression analyses clearly demonstrated that the heterozygous and homozygous deletion of the *lgr4* gene resulted in a concomitant reduction of *lgr4* mRNA (Supplemental Figure 5, E and D).

#### Live-cell imaging of GnRH3 fibers in lgr4 KD and KO embryos.

To assess the role of lgr4 during GnRH3 fibers development, lgr4 KD (morphants) and lgr4 KO (Crispants) embryos were anesthetized with tricaine, embedded at 48 or 72 hpf in UltraPure Low Melting Point Agarose (Thermo Fisher Scientific), and analyzed using a confocal laser scanning microscope (Nikon C2+) with a ×20 objective. GnRH3 fiber structure was assessed using ImageJ software (NIH). Owing to the complexity of GnRH3 fibers, a specific region of interest (ROI) was selected and analyzed at each developmental stage, with background fluorescence subtracted from each image. The number of green pixels within each ROI was used as a proxy for the amount of GnRH3 fibers. Given the previous characterization of KD and KO strategies, after confocal acquisition at 48 and 72 hpf, the total RNA was extracted from single embryos (Std_Ctrls and morphants, and uninjected ctrls and Crispants) and the expression of *lgr4* was confirmed by qPCR as previously described.

### Statistics

For all experiments, data are shown as mean ± SEM. To determine statistical significance for parametric tests, the unpaired 2-tailed *t* test or, for multiple comparisons, 1-way ANOVA was used. Nonparametric tests were used when the data did not follow a specific distribution. In this case, for multiple comparison, the Kruskal-Wallis test was used. *P* values less than 0.05 and 0.01 were considered statistically significant. A *P* value less than 0.001 was considered highly significant. The statistical analysis was performed using GraphPad Prism7 (GraphPad Software).

### Study approval

#### Patients.

Written informed consent was obtained from all participants. The study protocol was approved by the Ethics Committee for Pediatrics, Adolescent Medicine and Psychiatry, Hospital District of Helsinki and Uusimaa (570/E7/2003). UK ethical approval was granted by the London-Chelsea NRES committee (13/LO/0257). The study was conducted in accordance with the guidelines of The Declaration of Helsinki.

#### Animal studies.

The study was carried out in accordance with the recommendation of the Local Ethical Committee of Université Libre de Bruxelles, and experimental procedures were approved under the Ethical Protocol 534N. All zebrafish husbandry and all experiments were performed under standard conditions in accordance with institutional (University of Milan) and Italian national ethical and animal welfare guidelines and regulations.

## Author contributions

LD organized collection of the patient cohort. LD, LG, SRH, and AM conceived of the study. AM, LG, MIG, ML, AL, RO, and IH performed experiments. FM performed the zebrafish study. VV and MB supervised the zebrafish study. AM, LG, LD, AC, KW, FM, MB, and SRH analyzed the data. AD and MJS carried out the in silico analysis. CPC and MRB assisted with the bioinformatics analysis of the data. GV provided financial support for the Lgr4 KO mouse model. LD, LG, AC, and SRH supervised the research. SRH, AM, FM, and LG wrote the manuscript. Co-first authorship was assigned to AM in view of her principal role in carrying out in vitro experiments, as well as her role in conceiving and coordinating the study, analyzing the data, and writing the manuscript. SH was assigned second co-first author in view of her principal role in gene discovery, coordinating and analyzing data, and supervision and manuscript preparation. All authors discussed, commented on, and approved the final version of the manuscript.

## Supplementary Material

Supplemental data

## Figures and Tables

**Table 1:**
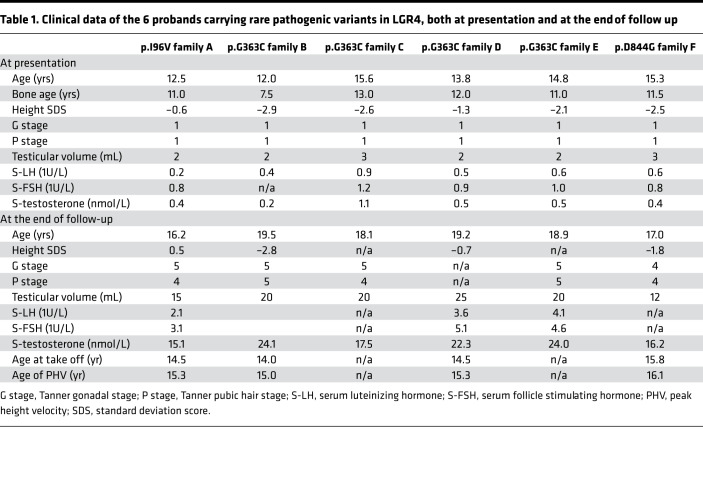
Clinical data of the 6 probands carrying rare pathogenic variants in LGR4, both at presentation and at the end of follow up

## Version 1 06/04/2020

Electronic publication

## References

[1] Howard SR (2016). IGSF10 mutations dysregulate gonadotropin-releasing hormone neuronal migration resulting in delayed puberty. EMBO Mol Med.

[2] Howard SR (2018). HS6ST1 insufficiency causes self-limited delayed puberty in contrast with other GnRH deficiency genes. J Clin Endocrinol Metab.

[3] Elks CE (2010). Thirty new loci for age at menarche identified by a meta-analysis of genome-wide association studies. Nat Genet.

[4] Styrkarsdottir U (2013). Nonsense mutation in the LGR4 gene is associated with several human diseases and other traits. Nature.

[5] Glinka A (2011). LGR4 and LGR5 are R-spondin receptors mediating Wnt/β-catenin and Wnt/PCP signalling. EMBO Rep.

[6] Zhu J, Chan YM (2017). Adult consequences of self-limited delayed puberty. Pediatrics.

[7] Day FR (2017). Genomic analyses identify hundreds of variants associated with age at menarche and support a role for puberty timing in cancer risk. Nat Genet.

[8] Palmert MR, Dunkel L (2012). Clinical practice. Delayed puberty. N Engl J Med.

[9] Wehkalampi K, Widén E, Laine T, Palotie A, Dunkel L (2008). Patterns of inheritance of constitutional delay of growth and puberty in families of adolescent girls and boys referred to specialist pediatric care. J Clin Endocrinol Metab.

[10] Howard SR (2018). Contributions of function-altering variants in genes implicated in pubertal timing and body mass for self-limited delayed puberty. J Clin Endocrinol Metab.

[11] Mancini A (2019). EAP1 regulation of GnRH promoter activity is important for human pubertal timing. Hum Mol Genet.

[12] Van Schoore G, Mendive F, Pochet R, Vassart G (2005). Expression pattern of the orphan receptor LGR4/GPR48 gene in the mouse. Histochem Cell Biol.

[13] Carmon KS, Gong X, Lin Q, Thomas A, Liu Q (2011). R-spondins function as ligands of the orphan receptors LGR4 and LGR5 to regulate Wnt/β-catenin signaling. Proc Natl Acad Sci U S A.

[14] Bowman AN, van Amerongen R, Palmer TD, Nusse R (2013). Lineage tracing with Axin2 reveals distinct developmental and adult populations of Wnt/β-catenin-responsive neural stem cells. Proc Natl Acad Sci U S A.

[15] Yi J, Xiong W, Gong X, Bellister S, Ellis LM, Liu Q (2013). Analysis of LGR4 receptor distribution in human and mouse tissues. PLoS One.

[16] Messina A (2016). A microRNA switch regulates the rise in hypothalamic GnRH production before puberty. Nat Neurosci.

[17] Abraham E, Palevitch O, Gothilf Y, Zohar Y (2009). The zebrafish as a model system for forebrain GnRH neuronal development. Gen Comp Endocrinol.

[18] Bassi I (2016). The zebrafish: an emerging animal model for investigating the hypothalamic regulation of reproduction. Minerva Endocrinol.

[19] Cariboni A (2015). Dysfunctional SEMA3E signaling underlies gonadotropin-releasing hormone neuron deficiency in Kallmann syndrome. J Clin Invest.

[20] Howard SR, Dunkel L (2019). Delayed puberty-phenotypic diversity, molecular genetic mechanisms, and recent discoveries. Endocr Rev.

[21] Bonomi M (2018). Characteristics of a nationwide cohort of patients presenting with isolated hypogonadotropic hypogonadism (IHH). Eur J Endocrinol.

[22] Carmon KS, Gong X, Yi J, Thomas A, Liu Q (2014). RSPO-LGR4 functions via IQGAP1 to potentiate Wnt signaling. Proc Natl Acad Sci U S A.

[23] Hao HX, Jiang X, Cong F (2016). Control of Wnt receptor turnover by R-spondin-ZNRF3/RNF43 signaling module and its dysregulation in cancer. Cancers (Basel).

[24] Sedlmeyer IL, Hirschhorn JN, Palmert MR (2002). Pedigree analysis of constitutional delay of growth and maturation: determination of familial aggregation and inheritance patterns. J Clin Endocrinol Metab.

[25] Freese JL, Pino D, Pleasure SJ (2010). Wnt signaling in development and disease. Neurobiol Dis.

[26] Silveira LG (2010). Mutations of the KISS1 gene in disorders of puberty. J Clin Endocrinol Metab.

[27] Herbison AE, Porteous R, Pape JR, Mora JM, Hurst PR (2008). Gonadotropin-releasing hormone neuron requirements for puberty, ovulation, and fertility. Endocrinology.

[28] Colledge WH, Mei H, d’Anglemont de Tassigny X (2010). Mouse models to study the central regulation of puberty. Mol Cell Endocrinol.

[29] d’Anglemont de Tassigny X (2007). Hypogonadotropic hypogonadism in mice lacking a functional Kiss1 gene. Proc Natl Acad Sci U S A.

[30] Hoshii T, Takeo T, Nakagata N, Takeya M, Araki K, Yamamura K (2007). LGR4 regulates the postnatal development and integrity of male reproductive tracts in mice. Biol Reprod.

[31] Mendive F, Laurent P, Van Schoore G, Skarnes W, Pochet R, Vassart G (2006). Defective postnatal development of the male reproductive tract in LGR4 knockout mice. Dev Biol.

[32] Moreira-Andrés MN, Cañizo FJ, de la Cruz FJ, Gómez-de la Cámara A, Hawkins FG (1998). Bone mineral status in prepubertal children with constitutional delay of growth and puberty. Eur J Endocrinol.

[33] Finkelstein JS, Neer RM, Biller BM, Crawford JD, Klibanski A (1992). Osteopenia in men with a history of delayed puberty. N Engl J Med.

[34] Crowne EC, Shalet SM (1990). Management of constitutional delay in growt and puberty. Trends Endocrinol Metab.

[35] Kaltiala-Heino R, Kosunen E, Rimpelä M (2003). Pubertal timing, sexual behaviour and self-reported depression in middle adolescence. J Adolesc.

[36] Day FR, Elks CE, Murray A, Ong KK, Perry JR (2015). Puberty timing associated with diabetes, cardiovascular disease and also diverse health outcomes in men and women: the UK Biobank study. Sci Rep.

[37] The UniProt Consortium (2017). UniProt: the universal protein knowledgebase. Nucleic Acids Res.

[38] Berman HM (2000). The Protein Data Bank. Nucleic Acids Res.

[39] Kelley LA, Mezulis S, Yates CM, Wass MN, Sternberg MJ (2015). The Phyre2 web portal for protein modeling, prediction and analysis. Nat Protoc.

[40] Schymkowitz J, Borg J, Stricher F, Nys R, Rousseau F, Serrano L (2005). The FoldX web server: an online force field. Nucleic Acids Res.

[41] Kumar P, Henikoff S, Ng PC (2009). Predicting the effects of coding non-synonymous variants on protein function using the SIFT algorithm. Nat Protoc.

[42] Adzhubei IA (2010). A method and server for predicting damaging missense mutations. Nat Methods.

[43] Leighton PA (2001). Defining brain wiring patterns and mechanisms through gene trapping in mice. Nature.

[44] Guasti L, Paul A, Laufer E, King P (2011). Localization of Sonic hedgehog secreting and receiving cells in the developing and adult rat adrenal cortex. Mol Cell Endocrinol.

[45] Cariboni A (2011). VEGF signalling controls GnRH neuron survival via NRP1 independently of KDR and blood vessels. Development.

[46] Abraham E, Palevitch O, Ijiri S, Du SJ, Gothilf Y, Zohar Y (2008). Early development of forebrain gonadotrophin-releasing hormone (GnRH) neurones and the role of GnRH as an autocrine migration factor. J Neuroendocrinol.

[47] Westerfield M. *The zebrafish book. A guide for the laboratory use of zebrafish (Danio rerio)*. Eugene, Oregon, USA: University of Oregon Press; 2000.

[48] Kimmel CB, Ballard WW, Kimmel SR, Ullmann B, Schilling TF (1995). Stages of embryonic development of the zebrafish. Dev Dyn.

[49] Jobst-Schwan T (2018). Acute multi-sgRNA knockdown of KEOPS complex genes reproduces the microcephaly phenotype of the stable knockout zebrafish model. PLoS One.

