# Supplementary material for: LGR4 deficiency results in delayed puberty through impaired Wnt/β-catenin signaling
Source: JCI Insight. 2020 Jun 4;5(11):e133434. doi: 10.1172/jci.insight.133434 (PMC7308048; doi:10.1172/jci.insight.133434)

Supplemental material

LGR4 deficiency results in delayed puberty through impaired Wnt-β-catenin signaling

Authors – Alessandra Mancini[1§], Sasha R Howard[1§], Federica Marelli[2,3], Claudia P Cabrera[4,5], Michael R Barnes[4,5], Michael JE Sternberg[6], Morgane Leprovots[7], Irene Hadjidemetriou[1], Elena Monti[8], Alessia David[6], Karoliina Wehkalampi[9], Roberto Oleari[10], Antonella Lettieri[10], Valeria Vezzoli[3], Gilbert Vassart[7], Anna Cariboni[10], Marco Bonomi[2,3], Marie Isabelle Garcia[7]*, Leonardo Guasti[1]*, Leo Dunkel[1]*

*co-senior author §co-first author

Supplemental Methods

Zebrafish experiments: We used *tg*(*gnrh3*:gfp) embryos for confocal live imaging to characterize changes in GnRH3-neuron morphology during zebrafish development. The GnRH3 system starts to develop around 24 hpf from cells located in the olfactory epithelium and, at 48 and 72 hpf, they elongate caudoventrally along the pre-optic area with extensions reaching the hypothalamus [1].

Lgr4 knockdown by morpholino microinjection:Morpholinos were dissolved in Danieau's solution (58 mMNaCl; 0,7 mMKCl; 0,4 mM MgSO4.H2O; 0,6 mMCa(NO3)2; 5 mMHepes pH 7.2) at 2 mM and stored at –80°C. Embryos were microinjected at the 1–4 cell stage embryos with rhodamine dextran (Molecular Probes) co-injected as a tracer. Morpholinos were tested for efficacy and toxicity by injecting different doses in *tg*(*gnrh3*:gfp) embryos (referred to as Morphants) and evaluating them for the presence of morphological defects (**Supplementary Figure S5-A** to **C**). Lgr4 Morphants were compared with embryos injected with 1.25 pmol/e of a control MO (Std_Ctrl).

The total RNA was extracted for pools of 30 injected embryos per group using Trizol Reagent (Life Technologies) and 1µg of RNA was retrotranscribed using GoScript Reverse Transcription System (Promegam Madison) following manufacturer's instruction. RT-PCR was assessed using the HotStartGotTaq and primers designed on the exon 1/2 and exon 3/4 boundaries of lgr4 cDNA (Fw: GGCATTAGGTTGAAGGAACTA; Rw: TCATTACCAGCCAAACGTAG), and primers for β-actin (Fw: CGAGCAGGAGATGGGAACC; Rw: CAACGGAAACGCTCATTGC) as endogenous control. qPCR was performed using SyberGreenMasterMix (Life Technologies) and primers designed on the exon 15/16 and 17/18 boundaries (Fw: TAACCTGGATCTCAGTCTCA; Rw: GTTATGGACCTGCGTTTAGG). The ddCT method and eef1a as an internal control were used to analyse the relative lgr4 expression.

Lgr4 knockout using the CRISPR/Cas9 System: Stimulated by the high efficiency of the CRISRR/Cas9 System to generate monoallelic and biallelic gene disruption in F0 zebrafish

embryos [1,2,3,4], we combined optimized mutagenesis conditions with a phenotyping strategy using the *tg*(*gnrh3*:gfp) line. The method displaying the highest mutagenesis efficiency is based on microinjection of an *in vitro* pre-assembled complex of guide RNA and Cas9 protein in one-cell stage embryos [2,5]. The F0 injected larvae, carrying mosaic loss-of-function (LOF) mutations, can be directly phenotyped and used to study function of candidate genes, a strategy known as transient knockdown approach [1,2,5,6]. This method permits a rapid and sensitive detection of GnRH phenotypes related to *lgr4* defects in live fish. Given the activation of non-homologous end-joining (NHEJ) repair system after Cas9-mediated double-strand break on the target DNA sequences we can presume that the F0 injected embryos carried a somatic mosaic of LOF mutations. Moreover, the resulted genetic KO implies the absence of mutated transcripts that has been suggested to activate the genetic compensation in zebrafish that can produce false-negative results [7].

Single guide RNA (sgRNA) targets were selected using the CHOPCHOP online tool v1 (https://chopchop.rc.fas.harvard.edu) following their ranking algorithm that takes into account all potential off-target sites differing in up to 2 nucleotides, and other factors such as GC content and the presence of protospacer adjacent motif (PAM) sequence. Since gRNA activity is higher if two guanine bases follow the T7 promoter, template sequences were modified accordingly if necessary. 2 µl of each sgRNA stock (250 ng/ul) were mixed with 1 µl of recombinant Cas9 protein (375 ng/µl, PNA Bio, Thousand Oaks, CA) and incubated on ice for at least 10 min to allow formation of the sgRNA/Cas9 complex. 4 nl of the sgRNAs-Cas9 mix was injected intracellularly in one-cell stage embryos (referred to as Crispants) using glass needles and a micromanipulator.

After morphological evaluation, to test the introduction of the desired deletion, the genomic DNA and the total RNA were extracted from the head or trunk/tail of single injected embryos and uninjected controls at 24hpf, using standard protocols. Mutagenesis was analysed by PCR amplification of the DNA using primers flanking the sites targeted by the sgRNAs (Fw:

CTATCCTGCAGAGAGGGAGAGA; Rw: GGCATGCACTTGATTGTTAAAG) followed by gel electrophoresis to discriminate the WT and the mutated amplicons.

Additional fluorescent expression studies: *In situ* hybridization (ISH) was performed on paraformaldehyde (PFA)-fixed cryosections from wild type C57BL/6 animals incubated with digoxigenin (DIG)-labelled anti-sense riboprobes for mouse *Lgr4*, as described in the main text. Immunostaining for GnRH was performed subsequently using the standard protocol described in the main text. Sections were incubated overnight at 4°C with rabbit anti-GnRH (1:400; Immunostar) and 2 hours with secondary antibody 488-conjugated donkey anti-rabbit IgG (1:400; Jackson Immunoresearch).Nuclei were counterstained with DAPI (1:10000; Sigma-Aldrich).

Image acquisition: ISH images were inverted and red channel was used to superimpose it on green channel, used for immunostaining.

Supplemental figures

A

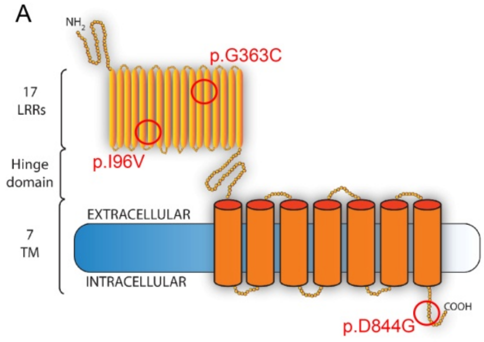

C

	Prediction software tools					
LGR4 variants	SIFT	PolyPhen2	CADD	Mutation Assessor	Mutation Taster	MAF(%) Finnish/European/All
p.I96V	0.09	0.077	22	0.234	Disease causing	0.04/0.01/0.008
p.G363C	0.11	0.996	25	0.728	Disease causing	1.50/1.10/1.44
p.D844G	0.01	0.996	33	0.611	Disease causing	0.18/1.92/1.21

B

	p.I96V	p.G363C	p.D844G
Human	TTTGGGTGGATAAAAGAAAGG	TTCTTCCAGAGCATGGCAACC	CATGCCACAGTCGTAGTAGAA
Chimpanzee	TTTGGGTGGATAAAAGAAAGG	TTCTTCCAGAGCATGGCAACC	CATGCCACAGTCGTAGTAGAA
Gorilla	TTTGGGTGGATAAAAGAAAGG	TTCTTCCAGAGCATGGCAACC	CATGCCACAGTCGTAGTAGAA
Orangutan	TTTGGGTGGATAAAAGAAAGG	TTCTTCCAGAGCATGGCAACC	CATGCCACAGTCGTAGTAGAA
Macaque	TTTGGGTGGATAAAAGAAAGG	TTCTTCCAGAGCATGGCAACC	CATGCCACAGTCGTAGTAGAA
Marmoset	TTTGGGTGGATAAAAGAAAGG	TTCTTCCAGAGCATGGCAACC	CATGCCACAGTCATAGTAGAA
Mouse	TTTGGGTGGATAAAAGAAAGG	TTCTTCCAATGCACGACAACC	CATGCCACAGTCGTAGTAGAA
Rat	TTTGGGTGGATAAAAGAAAGG	TTCTTCCAATGCACGACAACC	CATGCCACAGTCATAGTAGAA
Rabbit	TTTGGGTGGATAAAAGAAAGG	TTCTTCCAGAGCATGGCAACC	CATGCCACAGTCATAATAGAA
Cow	TTTGGGTGGATAAAAGAAAGG	TTCTTCCAGAGCATGGCAACC	CATGCCGCAGTCATAGTAGAA
Sheep	TTTGGGTGGATAAAAGAAAGG	TTCTTCCAGAGCATGGCAACC	CATGCCGCAGTCGTAGTAGAA
Pig	TTTGGGTGGATAAAAGAAAGG	TTCTTCCAGAGCATAGCAACC	CATGCCACAGTCATAGTAGAA
Dog	TTTGGGTGGATAAAAGAAAGG	TTCTTCCAGAGCATGGCAACC	CATGCCACAGTCATAGTAGAA
Cat	TTTGGGTGGATAAAAGAGAGG	TTCTTCCAGAGCATGGCAACC	CATGCCACAGTCATAGTAGAA
Horse	TTTGGGTGGATAAAAGAAAGG	TTCTTCCAGAGCATGGCAACC	CATGCCACAGTCGTAGTAGAA

Figure S1: LGR4 variants are highly conserved and predicted deleterious.

A) Representative LGR4 protein structure with representative localisation of missense variants. **B)**Multiple sequence alignment showing highly conserved nucleotides among species. Genomic Evolutionary Rate Profiling (GERP) score: p.I96V=6.71; p.G363C=3.95; p.D844G=3.80; scores have been retrieved using Ensembl. **C)** Prediction of pathogenicity summaryaccording to web-based prediction software programs: values in green represent scores for variant predicted to be *benign*, values in yellow, *possibly damaging/possibly deleterious* and values in red, *deleterious*. Minor allele frequency (MAF) data for the Finnish, European and other populations were retrieved from ExAC browser.

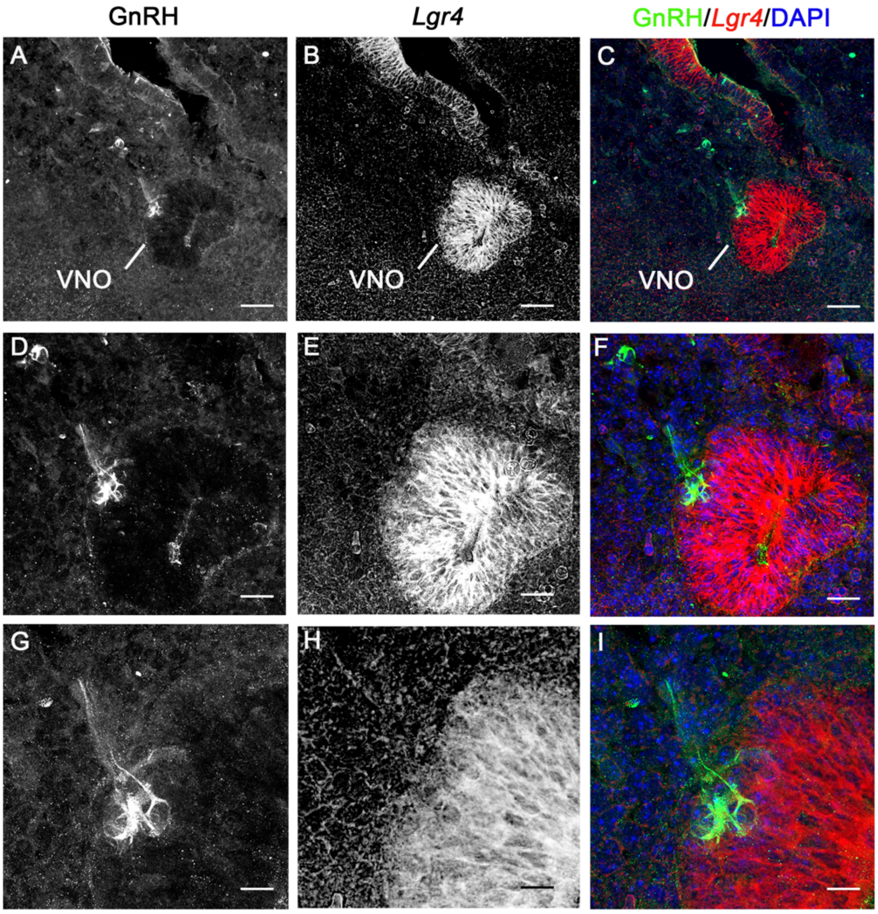

Figure S2: Native GnRH neurons exit from *Lgr4* positive VNO cells at E12.5.

A) Immunofluorescence detected GnRH neurons exiting the VNO at E12.5 and **B)***Lgr4* was strongly detected using *in situ* hybridization within the VNO. **C)** Merged image of A) and B). **D)**, **E)** and **F)** images at higher magnification. **G)**, **H)** and **I)** show one particular GnRH neuron migrating outside the Lgr4 positive VNO. Scale bars: A-C 125um, D-F 50um, G-I 25um.

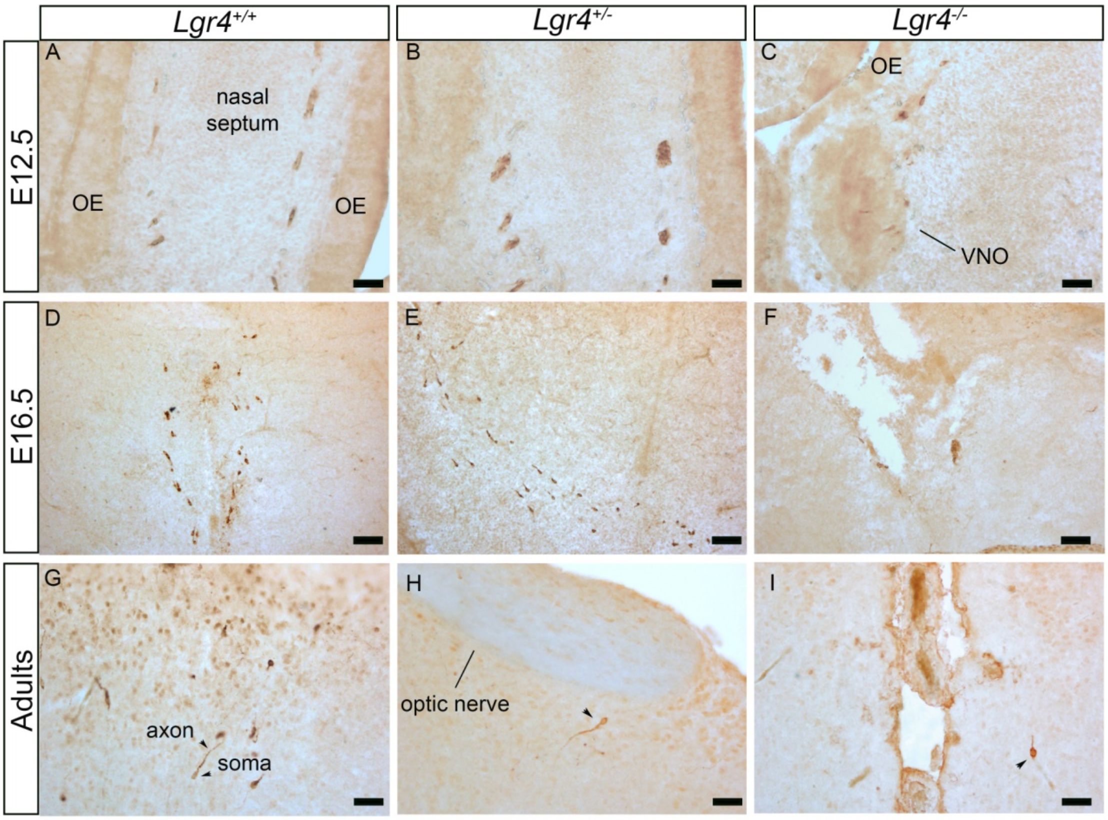

Figure S3: GnRH neuronal morphologyappears unchanged between *Lgr4*$^{+/+}$, *Lgr4*$^{+/-}$ and *Lgr4*$^{-/-}$ mice. **A)***Lgr4*$^{+/+}$,**B)***Lgr4*$^{+/-}$and **C)***Lgr4*$^{-/-}$mice at E12.5;**D)***Lgr4*$^{+/+}$,**E)***Lgr4*$^{+/-}$and **F)***Lgr4*$^{-/-}$mice at E16.5; **G)***Lgr4*$^{+/+}$,**H)***Lgr4*$^{+/-}$and **i)***Lgr4*$^{-/-}$mice at adult stage. At all developmental stages GnRH neuronal morphological phenotype appears not affected by the genotype. OE: olfactory epithelium; VNO: vomeronasal organs; Scale bars: a)-b)-c) and g)-h)-i): 50 µm; d)-e)-f): 100 µm.

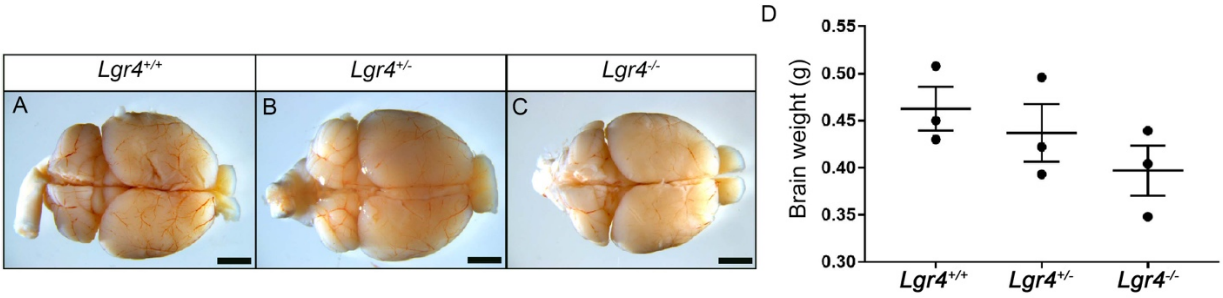

Figure S4: Gross anatomy of the brain reveals no major morphological differences between *Lgr4*$^{+/+}$, *Lgr4*$^{+/-}$ and *Lgr4*$^{-/-}$ mice. A), B) and C) Representative images of *Lgr4*$^{+/+}$,, *Lgr4*$^{+/-}$ and *Lgr4*$^{-/-}$ brains. **D)** Brains from *Lgr4*$^{+/-}$ mice have similar weight to *Lgr4*$^{+/+}$. *Lgr4*$^{-/-}$ mice have lower, but not significant brain weight, in line with lower total body weight. Scale bars: 2.5 mm

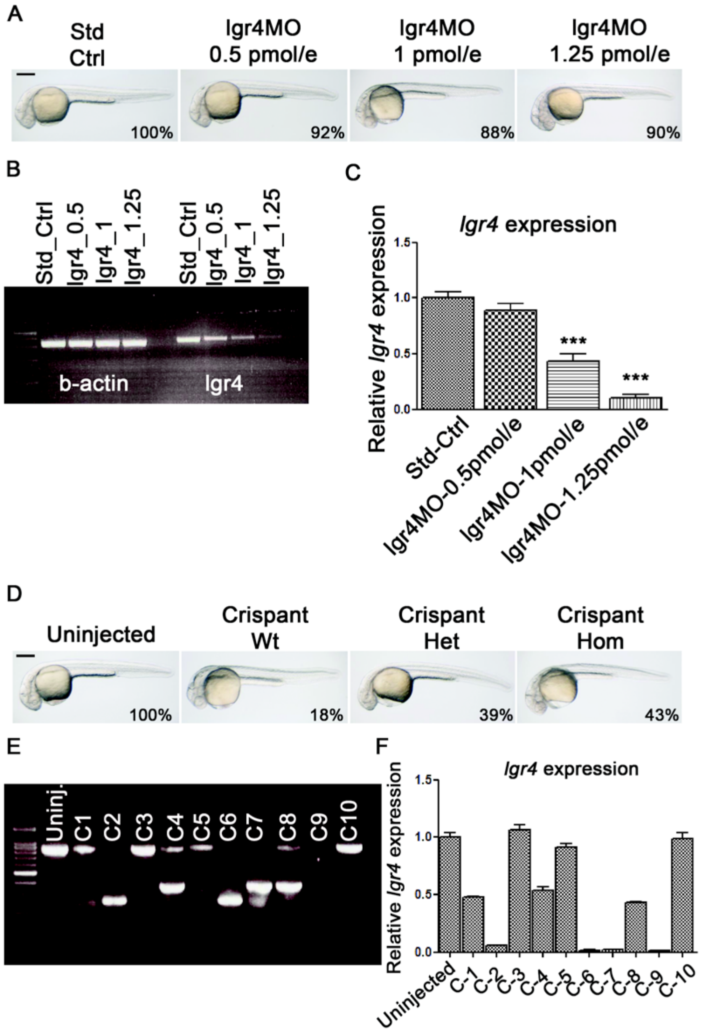

Figure S5: Morpholino and Crispr/Cas -mediated knockdown of the *lgr4* gene in zebrafish.A) Morphological evaluation of zebrafish development in embryos injected with the control MO (Std-Ctrls) and different doses (0.5, 1, and 1.25 pmol/e) of lgr4MO (Morphants) at 24 hpf. Images are acquired in lateral view, anterior to the left. Scale bar: 250 µm. Percentages on the images indicate rate of survival after injection. **B)** RT-PCR of WT lgr4 cDNA at 24 hpf, using primers designed on ex1/2 and ex2/3 boundaries, respectively. The b-actin was used as internal control.**C)**qRT-PCR of lgr4 expression at 24 hpf using primers designed on ex15/16 and ex17/18 boundaries, respectively. The relative mRNA expression was measured and normalised against the housekeeping gene *eef1a*. Each experiment was performed in triplicate using pools of 30 embryos/injection.**D)** Morphological evaluation of zebrafish development Crispants at 24 hpf, compared with the injected controls. Images are acquired in lateral view, anterior to the left. Percentages on the images indicate rate of successful monoallelic and biallelic inactivation of *lgr4* gene. Scale bar: 250 µm **E)** RT-PCR of *lgr4* genomic sequence using primers flanking the sgRNAs sites in controls and Crispants at 24 hpf. The upper band (873bp) correspond to the WT sequence, whereas the lower bands (<400bp) indicate the presence of mosaic somatic deletions. **F)** As previously described, qRT-PCR of *lgr4* expression at 24 hpf using primers designed on ex15/16 and ex17/18 boundaries, respectively. The relative mRNA expression was measured and normalised against the housekeeping gene *eef1a*. Results are expressed as mean and +/- SEM (***p<0.001). Each experiment was performed five times (10 embryo each), and the DNA and RNA samples were extracted from the head and trunk/tail regions of a single embryo.

References:

1. Trubiroha A*, et al.* A Rapid CRISPR/Cas-based Mutagenesis Assay in Zebrafish for Identification of Genes Involved in Thyroid Morphogenesis and Function. *Sci Rep***8**, 5647 (2018).

2. Burger A*, et al.* Maximizing mutagenesis with solubilized CRISPR-Cas9 ribonucleoprotein complexes. *Development***143**, 2025-2037 (2016).

3. Jao LE, Wente SR, Chen W. Efficient multiplex biallelic zebrafish genome editing using a CRISPR nuclease system. *Proceedings of the National Academy of Sciences of the United States of America***110**, 13904-13909 (2013).

4. Shah AN, Davey CF, Whitebirch AC, Miller AC, Moens CB. Rapid Reverse Genetic Screening Using CRISPR in Zebrafish. *Zebrafish***13**, 152-153 (2016).

5. Jobst-Schwan T*, et al.* Acute multi-sgRNA knockdown of KEOPS complex genes reproduces the microcephaly phenotype of the stable knockout zebrafish model. *PloS one***13**, e0191503 (2018).

6. Wu RS, Lam, II, Clay H, Duong DN, Deo RC, Coughlin SR. A Rapid Method for Directed Gene Knockout for Screening in G0 Zebrafish. *Developmental cell***46**, 112-125 e114 (2018).

7. Rossi A*, et al.* Genetic compensation induced by deleterious mutations but not gene knockdowns. *Nature***524**, 230-233 (2015).

Full unedited gel for Figure 3-B (top: anti-HA; bottom: anti-GAPDH)

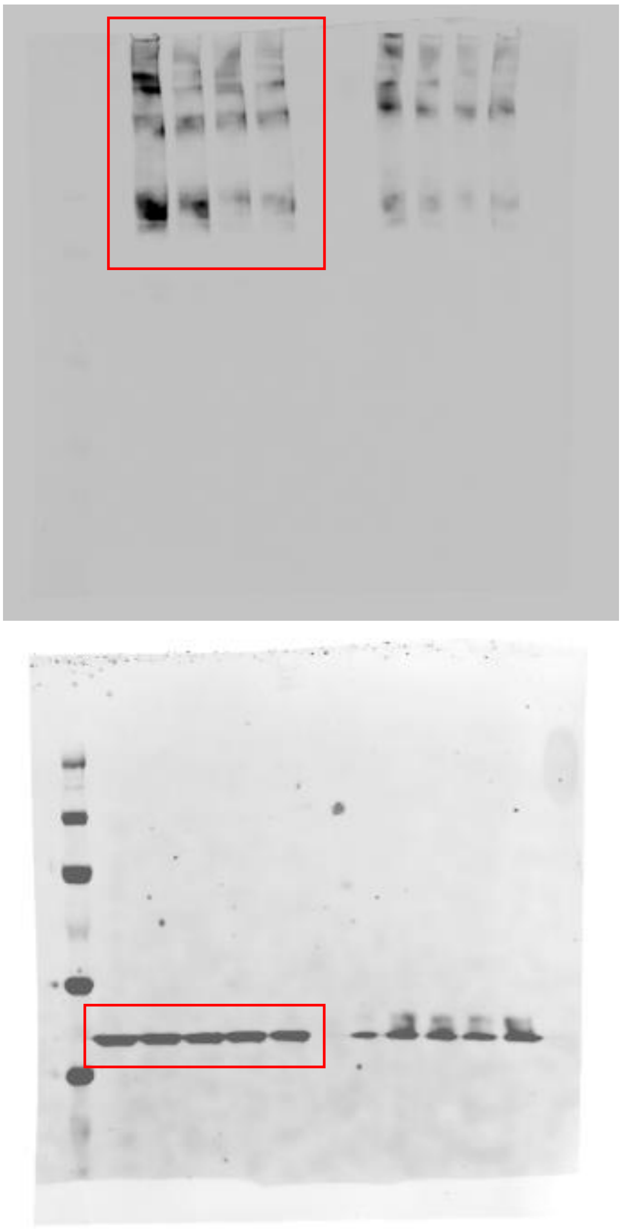

Full unedited gel for Figure 3-D (top: anti-HA; bottom: anti-b-actin)

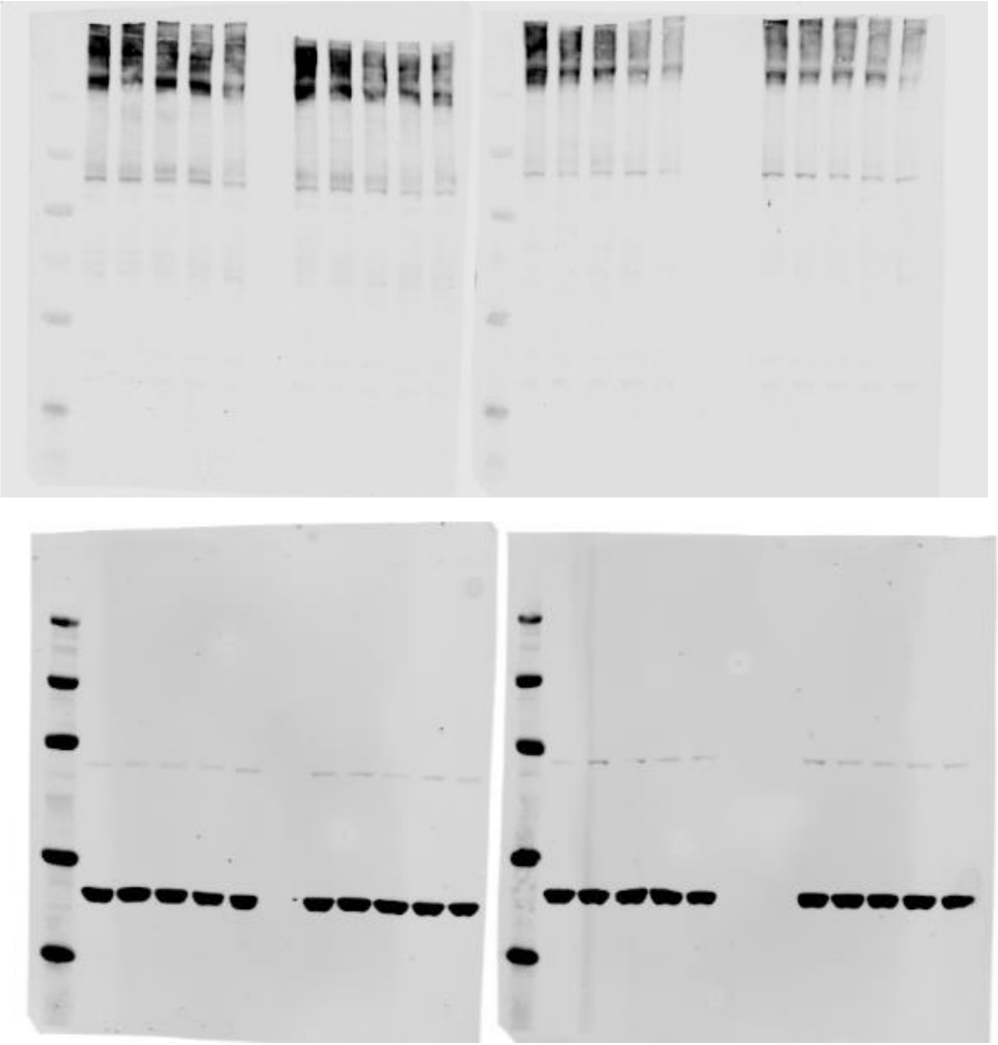

Supplement: Supplemental data [file jciinsight-5-133434-s163.pdf]
